# Development of a Novel ^18^F-Labeled Radioligand for Imaging Cholesterol 24-Hydroxylase with Positron Emission Tomography

**DOI:** 10.1021/acsptsci.4c00683

**Published:** 2025-02-24

**Authors:** Jian Rong, Chunyu Zhao, Ahmad F. Chaudhary, Jiahui Chen, Xin Zhou, Kuo Zhang, Zhendong Song, Zhenkun Sun, Yabiao Gao, Zachary Zhang, Siyan Feng, Thomas Lee Collier, Hongjie Yuan, Jimmy S. Patel, Achi Haider, Yinlong Li, Steven H. Liang

**Affiliations:** † Department of Radiology and Imaging Sciences, Emory University, Atlanta, Georgia 30322, United States; ‡ Department of Pharmacology and Chemical Biology, Emory University School of Medicine, Atlanta, Georgia 30322, United States

**Keywords:** cholesterol 24-hydroxylase, CYP46A1, cholesterol, positron emission tomography, PET, fluorine-18

## Abstract

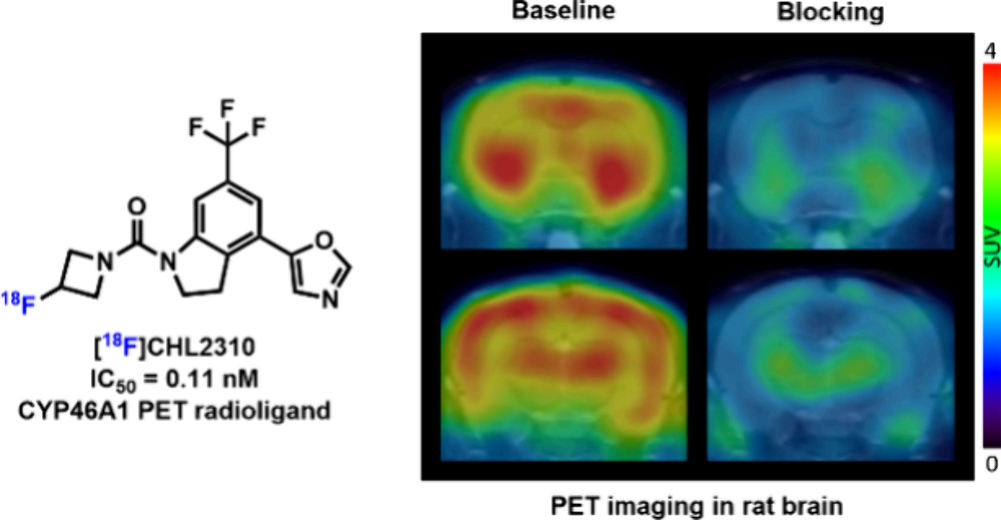

Cholesterol 24-hydroxylase (CYP46A1), also known as CH24H, is a brain-specific monooxygenase responsible for the elimination of cholesterol from the central nervous system (CNS). It catalyzes the conversion of cholesterol to 24(*S*)-hydroxycholesterol, the primary pathway for CNS cholesterol clearance. Dysregulation of cholesterol homeostasis has been implicated in neurodegenerative diseases, such as Alzheimer’s disease (AD) and Parkinson’s disease (PD). This study presents the synthesis and evaluation of [^18^F]**5** ([^18^F]CHL2310) as a novel radioligand for imaging CYP46A1 and cholesterol metabolism in the brain by positron emission tomography (PET). CHL2310 was identified as a potent inhibitor of CYP46A1 and subsequently labeled with fluorine-18 in a radiochemical yield of 13% and a high molar activity of 93 GBq/μmol. [^18^F]CHL2310 was evaluated in rats using in vitro autoradiography and PET imaging, demonstrating high brain uptake, heterogeneous brain distribution, favorable binding specificity, and suitable clearance kinetic profiles within the CNS. In all, [^18^F]**5** ([^18^F]CHL2310) represents a promising tool for noninvasive quantification of cholesterol metabolism by imaging CYP46A1.

As the principal sterol in animals, cholesterol plays a fundamental role not only in maintaining the structural integrity of cell membranes but also serves as a precursor for the biosynthesis of key molecules, including bile acids, steroid hormones, and vitamin D.^1^ However, the blood–brain barrier (BBB) restricts cholesterol’s movement between the CNS and peripheral tissues, thereby supporting an autonomous cholesterol homeostasis mechanism within the CNS.^2^ In the brain, cholesterol is synthesized de novo, mainly in glial cells and neurons.^3^ To maintain steady-state cholesterol levels, cholesterol elimination processes are essential. Cholesterol 24-hydroxylase (CYP46A1), a brain-specific cytochrome P450 monooxygenase, facilitates the conversion of cholesterol to 24(*S*)-hydroxycholesterol, which is capable of crossing the BBB and exiting the CNS.^4−6^ By mediating this conversion, CYP46A1 orchestrates the elimination of excess cholesterol from the CNS. Dysfunctions in cholesterol homeostasis have been broadly implicated in neurodegenerative diseases, including Alzheimer’s disease (AD), Parkinson’s disease (PD), and Huntington’s disease (HD).^7−11^ Along this line, CYP46A1 has emerged as a pivotal imaging target for exploring brain cholesterol dynamics and a potential therapeutic target for related disorders.

Positron emission tomography (PET) is a noninvasive medical imaging technique that can visualize biological processes at the molecular level in vivo.^12−14^ PET imaging of CYP46A1 has the potential to enable the assessment of CYP46A1 levels and visualization of cholesterol homeostasis in healthy and diseased brains. Despite recent progress, only a few CYP46A1 PET radioligands have been developed (Figure 1), including [^11^C]A,^15^ [^18^F]T-008,^16,17^ [^18^F]3g,^16^ and [^18^F]CHL2205.^18^ While [^11^C]A showed low brain uptake and marginal binding specificity in vivo, [^18^F]T-008, [^18^F]3g, and [^18^F]CHL2205 exhibited high brain uptake and good binding specificity toward CYP46A1. While [^18^F]T-008, [^18^F]3g, and [^18^F]CHL2205 have shown promising imaging characteristics, their structural similarities present a challenge in terms of developing structurally distinct alternatives with potentially improved pharmacokinetics and selectivity. The 4-indolinyl oxazole scaffold emerged as a promising framework due to the subnanomolar potency toward CYP46A1 and favorable lipophilicity for blood–brain barrier penetration.^19^ To introduce an ^18^F-labeling site while maintaining the integrity of the pharmacophore, we incorporated a 3-(fluoro-)azetidine moiety, leading to the design of CHL2310. This novel scaffold expands the available structural landscape for CYP46A1 PET radioligands, providing an opportunity to optimize imaging properties beyond what is currently available. Herein, we report the development of [^18^F]**5** ([^18^F]CHL2310) as a CYP46A1-specific PET radioligand bearing a novel 4-indolinyl oxazole scaffold for cholesterol metabolism imaging. With its high potency, compound **5** (CHL2310) was chosen for ^18^F-labeling, and the respective isotopologue was obtained in high radiochemical yield and molar activity. Further evaluations, including autoradiography and PET imaging studies in rats, demonstrated high brain uptake, heterogeneous brain distribution, and favorable binding specificity and clearance kinetics in the brain. These findings support [^18^F]**5** ([^18^F]CHL2310) as a promising CYP46A1-targeted PET radioligand for visualizing cholesterol metabolism in vivo, contributing to advancements in CNS disorders. Compared with previously developed CYP46A1 PET tracers, [^18^F]**5** exhibits several key advantages. Its 4-indolinyl oxazole scaffold represents a structurally distinct alternative to the piperidinyl pyrimidine-based ligands, broadening the chemical space for CYP46A1 imaging. Furthermore, [^18^F]**5** achieves subnanomolar affinity toward CYP46A1 (IC_50_ = 0.11 nM), comparable to or exceeding prior tracers in potency. Its high brain uptake (SUV_max_ = 4.2 in the thalamus) and strong target engagement are further supported by robust blocking studies. Moreover, we provide solid evidence from ex vivo experiments with [^18^F]**5** confirming that the brain signal stems primarily from the intact parent tracer. The absence of significant radiodefluorination and favorable biodistribution properties support its translational potential for CYP46A1-targeted imaging. Collectively, these attributes position [^18^F]**5** as a promising PET radioligand for CYP46A1, potentially offering superior imaging characteristics compared with existing alternatives. However, further evaluation in higher species is needed to rigorously assess its pharmacokinetic profile, metabolic stability, and suitability for clinical translation. These studies will be essential to confirm whether [^18^F]**5** outperforms previously reported CYP46A1 radioligands in humans.

**Figure 1:**
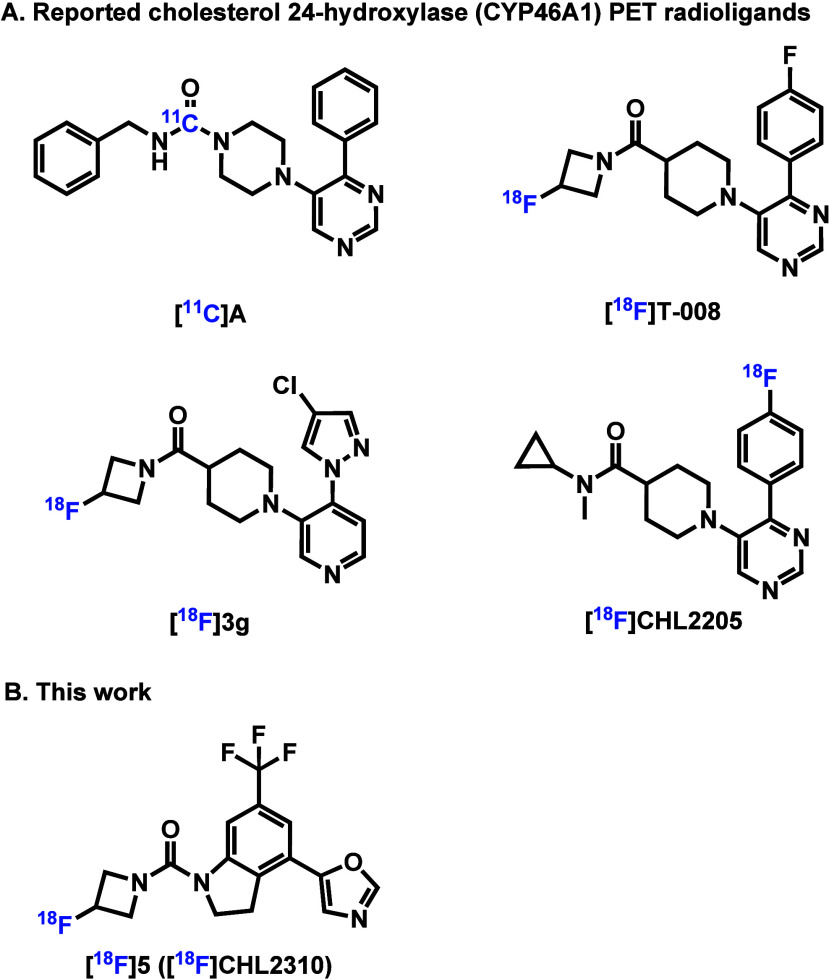
Representative cholesterol 24-hydroxylase (CYP46A1) PET radioligands.

## Results and Discussion

### Chemistry

**Scheme 1:**
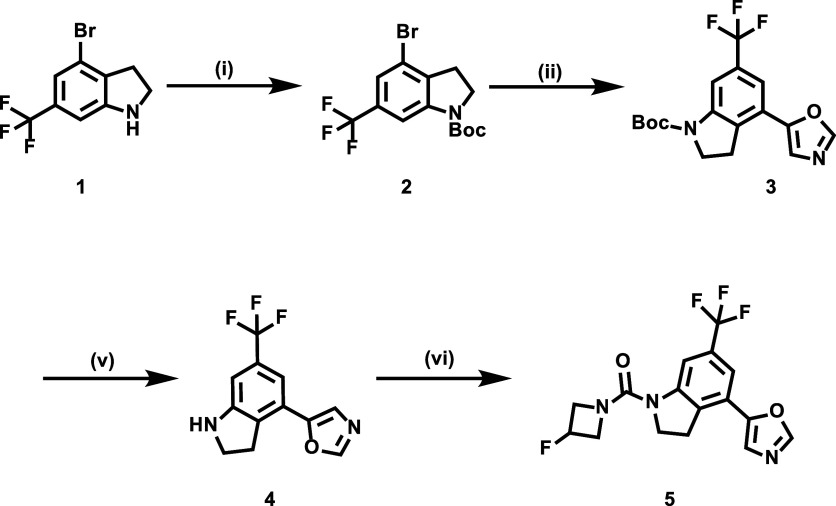
Synthesis of CYP46A1 Radioligand **5** Reaction conditions: (i) di-*tert*-butyl dicarbonate, NEt_3_, DCM, 0 to 25 °C, 12 h, 80% yield; (ii) Pd(dppf)Cl_2_, 5-(4,4,5,5-tetramethyl-1,3,2-dioxaborolan-2-yl)-2-[tris(1-methylethyl)silyl]oxazole, K_2_CO_3_, dioxane, N_2_, 85 °C, 16 h, 69% yield; (iii) trifluoroacetic acid, DCM, room temperature, 2 h; (iv) 3-fluoroazetidine-1-carbonyl chloride, NEt_3_, DCM, room temperature, 16 h, 11% yield. DCM = dichloromethane.

The development of compound **5** was informed by prior structure–activity relationship studies of CYP46A1 inhibitors, which identified the 4-indolinyl oxazole scaffold as a promising framework.^19^ To introduce an ^18^F-labeling site while maintaining strong CYP46A1 affinity, we incorporated a 3-(fluoro-)azetidine moiety, selected for its favorable pharmacokinetic properties and synthetic feasibility. This approach enabled us to maintain high potency while ensuring compatibility with PET imaging applications. The synthesis of compound **5** was successfully accomplished, starting from commercially available 4-bromo-6-(trifluoromethyl)indoline **1** (Scheme 1). Boc-protection of indoline **1** afforded compound **2** in 80% yield. The subsequent palladium-catalyzed cross-coupling reactions of compound **2** and 5-(4,4,5,5-tetramethyl-1,3,2-dioxaborolan-2-yl)-2-[tris(1-methylethyl)silyl]oxazole gave the trifluoromethyl derivative **3** in 69% yield. Deprotection of compound **3**, followed by a condensation reaction with 3-fluoroazetidine-1-carbonyl chloride, afforded target compound **5** in 11% yield over two steps.

### Pharmacology

**Figure 2:**
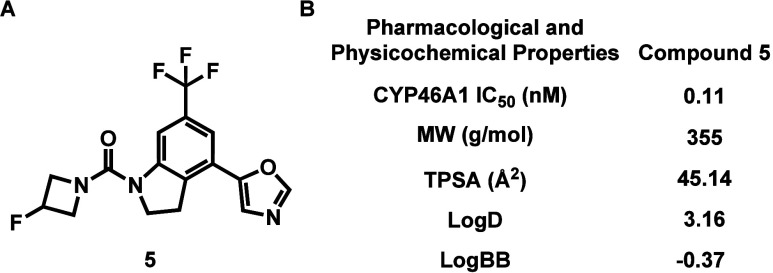
Pharmacological profile of compound **5**. (A) Chemical structure of **5**; (B) representative pharmacological and physicochemical properties of **5**.

Compound **5** exhibited excellent potency toward CYP46A1, with an IC_50_ of 0.11 nM (Figure 2). Its logD of 3.16 was measured by the shake flask method,^20^ indicating favorable physicochemical properties for compound **5**. The BBB permeability for compound **5** was predicted with ACD/Percepta, and the results (logBB = −0.37) suggested a high likelihood of CNS penetration (logBB > −1). Furthermore, off-target binding assays of compound **5** were performed in vitro against 67 major CNS targets (Figure S1 in the Supporting Information). There was no significant off-target binding (>50% inhibition) for compound **5** at 10 μM testing concentration, except for the sigma-2 receptor (*K*_*i*_ = 5421 nM).

### Radiochemistry

**Figure 3:**
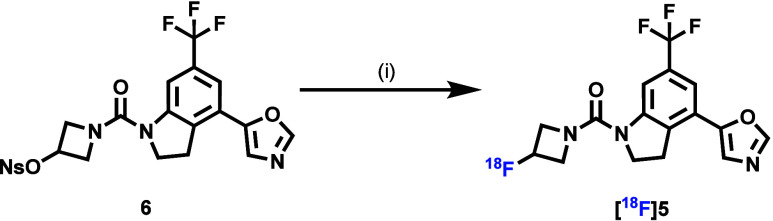
Radiolabeling of [^**18**^**F**]**5**. Reaction conditions: (i) [^18^F]Et_4_NF, DMF/*t*BuOH, 140 °C, 10 min, 13% decay-corrected RCY; DMF = dimethylformamide.

The ^18^F-labeling nosylate precursor **6** was prepared via a procedure similar to that of compound **5** in 31% yield over two steps (Scheme S1). As shown in Figure 3A, compound **5** was radiolabeled with fluorine-18 via nucleophilic substitution of the corresponding nosylate precursor with[^18^F]Et_4_NF in a mixture of DMF and *t*BuOH (v:v = 1:3) at 140 °C for 10 min. [^18^F]**5** was generated in a 13% decay-corrected radiochemical yield with a high molar activity of 93 GBq/μmol (2.5 Ci/ μmol). Additionally, [^18^F]**5** exhibited high in vitro stability, with no radiolysis detected in saline (18.5 MBq/mL, containing 5% of ethanol) for up to 60 min (Figure S2A). Furthermore, we investigated the stability of [^18^F]**5** in mouse, rat, NHP, and human serums, and minimal radiometabolite formation was observed in all serums up to 60 min (Figure S2B–E).

### In Vitro Autoradiography Study

**Figure 4:**
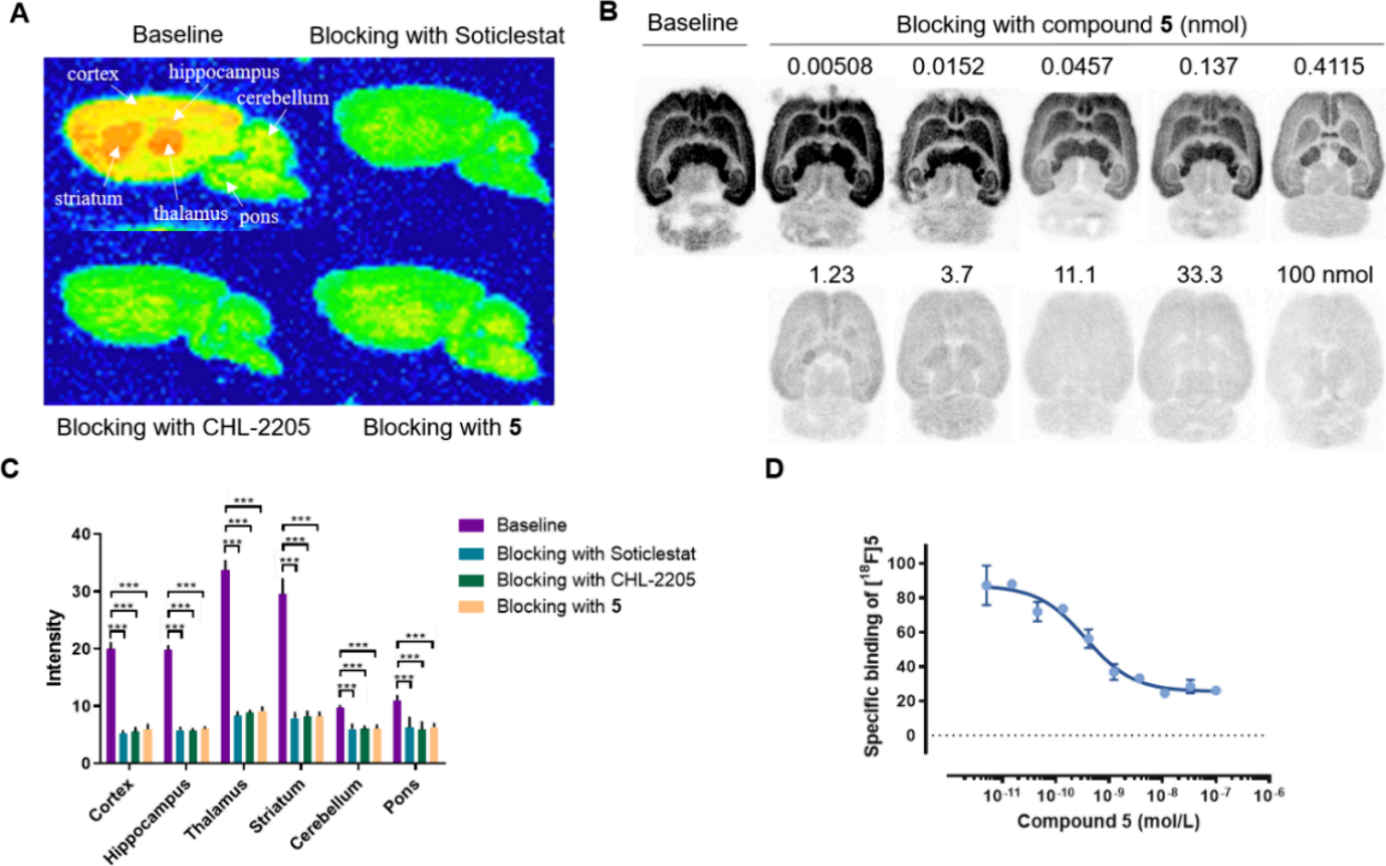
In vitro autoradiography study of [^18^F]**5** in the rat brain. (A) Representative images of baseline and blocking (Soticlestat, CHL-2205, or **5**, 10 μM) autoradiography studies with [^18^F]**5**; (B) representative images of baseline and blocking (**5**, in various concentrations) autoradiography studies with [^18^F]**5**; (C) quantification of autoradiography study of [^18^F]**5**; and (D) displacement curve of [^18^F]**5** with unlabeled **5** (*K*_d_ = 0.37 nM). All data are mean ± SD, *n* ≥ 3. Statistical analysis was calculated by one-way analysis of variance (ANOVA) test (***p ≤ 0.001).

To assess the binding specificity of [^18^F]**5** for CYP46A1, in vitro autoradiography was performed by using rat brain tissue sections. As shown in Figure 4A, the results demonstrated that [^18^F]**5** preferentially bound to the thalamus and striatum, followed by the cortex and hippocampus, with lower binding in the cerebellum and pons. The accumulation patterns are in agreement with the brain distribution of CYP46A1 in rodents.^18^ In blocking studies, the previously described CYP46A1 inhibitors, Soticlestat and CHL-2205,^18^ and compound **5** significantly diminished the radioactivity levels in CYP46A1-rich brain regions (73–75% reduction for the thalamus, 72–73% for the striatum, 70–74% for the cortex, and 69–71% for the hippocampus). These results demonstrated that [^18^F]**5** had a high in vitro binding specificity toward CYP46A1. Furthermore, [^18^F]**5** was utilized to assess the potency toward CYP46A1 through an inhibition assay based on an in vitro autoradiography study (Figure 4B). The assay was performed by the incubation of [^18^F]**5** on rat brain tissue sections in the presence of unlabeled **5** at varying concentrations. These findings corroborated a high potency toward CYP46A1, with a subnanomolar K_d_ value of 0.37 nM.

### PET Imaging Study

**Figure 5:**
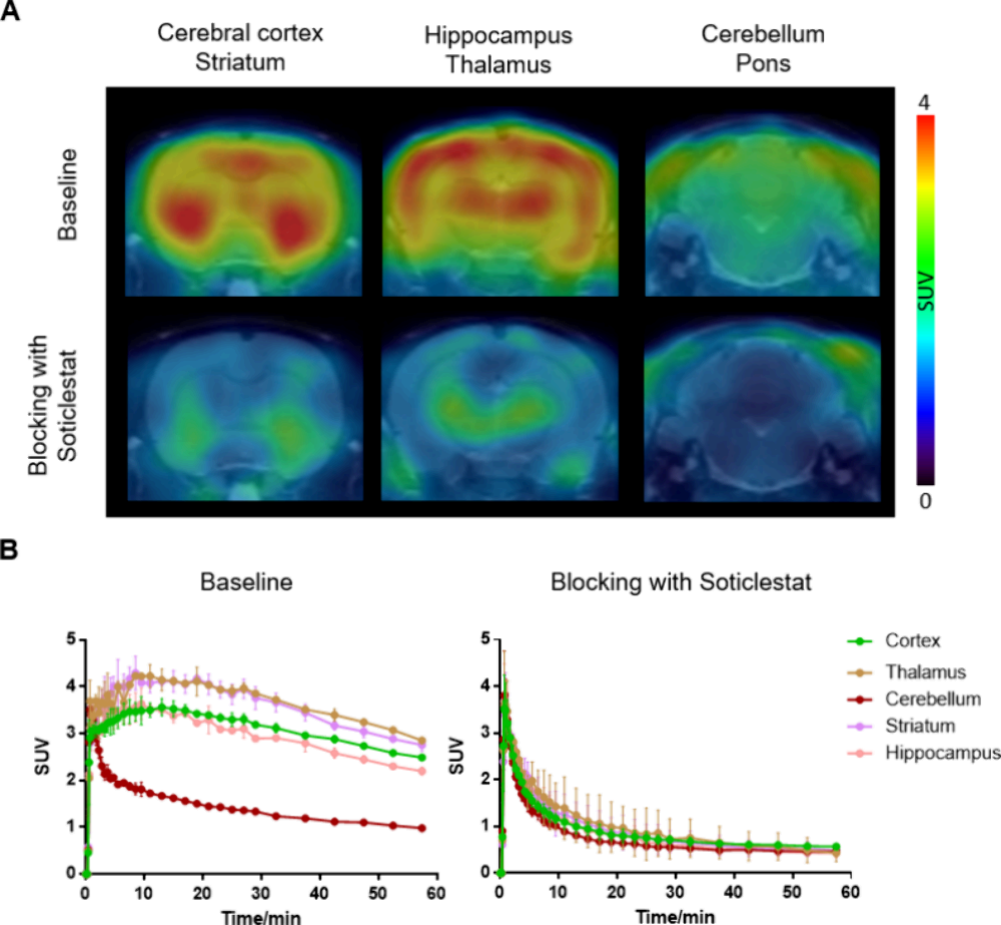
PET imaging study of [^18^F]**5** in the rat brain. (A) Representative summed PET images (0–60 min) of [^18^F]**5** under baseline and blocking (Soticlestat, 1 mg/kg) conditions, (B) TACs of [^18^F]**5** in brain regions of interest; all data are mean ± SD, *n* ≥ 3.

To investigate the in vivo binding specificity of [^18^F]**5** toward CYP46A1, dynamic PET imaging was performed in Sprague–Dawley (SD) rats (Figures 5 and S3 in the Supporting Information). [^18^F]**5** rapidly crossed the BBB and exhibited a heterogeneous brain distribution. High uptake values were found in the thalamus (SUV_max_ = 4.2) and striatum (SUV_max_ = 4.1), followed by the cortex and hippocampus, and low radioactivity levels were observed in the cerebellum and pons. The PET findings were in concert with the probe distribution patterns observed on in vitro autoradiograms of the rodent brain.^18^ [^18^F]**5** demonstrated high brain uptake in rodents, comparable to values reported for previously reported CYP46A1 PET radioligands. However, the early development stage of [^18^F]**5** and the lack of current data on higher species in the current study limit the scope of direct comparison. Notably, compound **5**’s 4-indolinyl oxazole scaffold provides an alternative structural framework to the piperidinyl pyrimidine class, potentially enhancing selectivity and metabolic stability. Future studies will be required to evaluate its suitability in higher species and direct comparisons under identical experimental conditions. In the blocking study, preadministration of Soticlestat significantly decreased the uptake of [^18^F]**5** (47–74% reduction across various brain regions defined by AUC for Soticlestat, 1 mg/kg). To investigate the reversibility of the tracer binding, we further performed a displacement study with Soticlestat at 20 min postadministration of [^18^F]**5** (Figure S4 in the Supporting Information). In the latter study, the brain signal was markedly reduced after the administration of Soticlestat, indicating that [^18^F]**5** had a high binding specificity toward CYP46A1 in vivo with a reversible binding mechanism, which would allow compartment model-based quantitative kinetic analysis in future studies.

### Whole-Body Biodistribution Study

**Figure 6:**
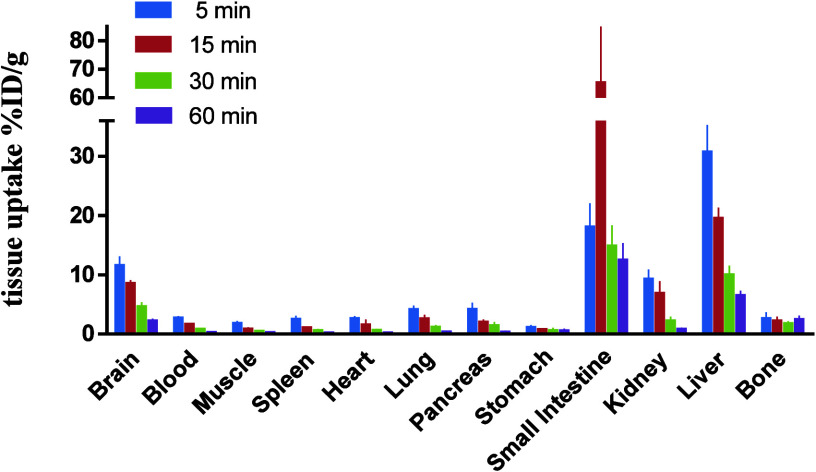
Whole-body biodistribution study of [^18^F]**5** in CD-1 mice. All data are mean ± SD, *n* = 3.

To investigate the distribution of [^18^F]**5** in the whole body, we conducted a biodistribution study in CD-1 mice (Figure 6). The radioactivity levels in major organs were assessed at 5, 15, 30, and 60 min after the administration of [^18^F]**5**. Initially, a relatively high uptake (around or above 10% ID/g) was observed in the brain, small intestine, kidney, and liver. Over time, the radioactive signal decreased in almost all organs, while the uptake in the small intestine increased, and liver uptake remained at a high level (6.5% ID/g at 60 min), indicating a hepatobiliary elimination route. No obvious increase in bone uptake was observed, suggesting negligible radiodefluorination of [^18^F]**5** in vivo.

### Radiometabolite Analysis

**Figure 7:**
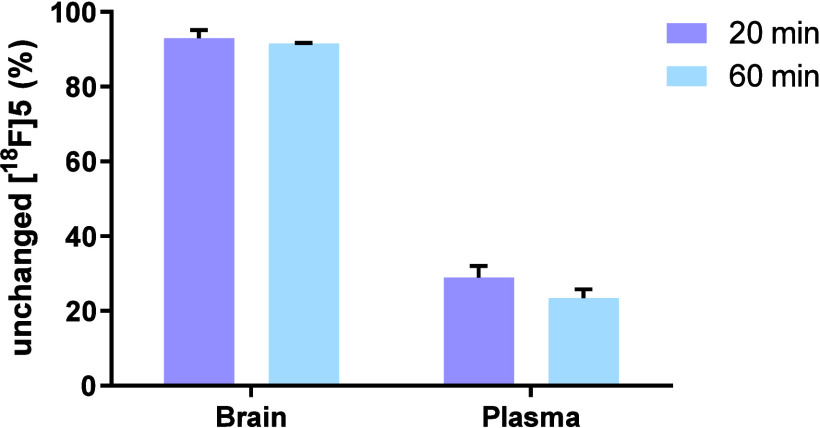
Radiometabolic analysis of [^18^F]**5** in SD rats. All data are mean ± SD, *n* = 3.

To gain insight into the in vivo stability of [^18^F]**5**, a radiometabolite analysis was conducted in SD rats (Figure 7). In the brain, 93 and 92% of [^18^F]**5** remained unchanged at 20 and 60 min postadministration of [^18^F]**5**, respectively. In the plasma, 29 and 23% of [^18^F]**5** remained unchanged at 20 and 60 min postadministration of [^18^F]**5**, respectively. The high stability of [^18^F]**5** in the brain indicated that the brain PET signal predominately originated from the parent compound [^18^F]**5**.

## Conclusions

Compound **5** (CHL2310) was designed by introducing the 3-(fluoro-)azetidine moiety as the labeling site into the promising 4-indolinyl oxazole scaffold. It was identified as a potent and selective CYP46A1 inhibitor and was successfully radiolabeled with fluorine-18, affording a reasonable radiochemical yield (13%, decay-corrected) and molar activity (93 GBq/μmol). [^18^F]**5** ([^18^F]CHL2310) was evaluated by in vitro autoradiography, PET imaging, ex vivo biodistribution studies, and radiometabolite analysis in rodents. These results revealed that [^18^F]**5** displayed heterogeneous brain distribution, in agreement with known CYP46A1 expression patterns as well as a high brain uptake (SUV > 4) with excellent binding specificity and brain kinetics. In summary, [^18^F]**5** exhibited promising characteristics as a suitable PET radioligand for imaging CYP46A1, and further evaluations are underway in higher species.

## Experimental Sections

### General Information

Chemicals were used directly in the synthesis as purchased. The NMR data were recorded by a 400 or 600 MHz NMR spectrometer. Coupling constants and chemical shifts were recorded in Hz and ppm, respectively. High-resolution mass data were collected in APCI mode. Animal studies were performed following the institutional ethical guidelines of the Institutional Animal Care and Use Committee (IACUC) of Emory University (PROTO202200003 and PROTO202200076). Rodents (SD rats: female, 8–10 weeks, 200–250 g; CD-1 mice: female, 6–8 weeks, 25–30 g) were fed under the 12h light/12h dark cycle.

### Chemistry

#### Synthesis of *Tert*-Butyl 4-bromo-6-(trifluoromethyl)indoline-1-carboxylate (**2**)

To a stirred solution of 4-bromo-6-(trifluoromethyl) indoline (200 mg, 0.75 mmol, 1.0 equiv) in dichloromethane (2 mL) at 0 °C, triethyl amine (227 mg, 2.25 mmol, 3 equiv) was added and stirred for 20 min. Then, di-*tert*-butyl dicarbonate (245 mg, 1.12 mmol, 1.5 equiv) was added and stirred at room temperature for 12 h. After that, the mixture was concentrated and purified by column chromatography to afford *tert*-butyl 4-bromo-6-(trifluoromethyl) indoline-1-carboxylate **2** (220 mg, 80% yield, off-white solid). ^1^H NMR (400 MHz, Chloroform-*d*) δ 8.05 (br, 1H), 7.33 (s, 1H), 4.05 (t, *J* = 8.8 Hz, 2H), 3.11 (t, *J* = 8.4 Hz, 2H), 1.57 (s, 9H). ^13^C NMR (100 MHz, Chloroform-*d*) δ 152.15, 144.57, 135.45, 131.72 (q, *J* = 31.6 Hz), 123.39 (q, *J* = 271.1 Hz), 121.97 (q, *J* = 4.2 Hz), 119.23 (q, *J* = 2.5 Hz), 110.39 (q, *J* = 3.9 Hz), 81.61, 47.31, 28.84, 28.31.

#### Synthesis of *Tert*-Butyl 4-(oxazol-5-yl)-6-(trifluoromethyl)indoline-1-carboxylate (**3**)

To a solution of *tert*-butyl 4-bromo-6-(trifluoromethyl)indoline-1-carboxylate **2** (135 mg, 0.37 mmol, 1 equiv) and 5-(4,4,5,5-tetramethyl-1,3,2-dioxaborolan-2-yl)-2-(triisopropylsilyl)oxazole (194 mg, 0.55 mmol, 1.5 equiv) in dioxane, K_2_CO_3_ (153 mg, 1.11 mmol, 3 equiv) and Pd(dppf)Cl_2_ (27 mg, 0.037 mmol, 0.1 equiv) were added under a N_2_ atmosphere and stirred at 85 °C for 16 h. Then, the mixture was concentrated and purified by column chromatography to afford product **3** (90 mg, 69%, yellow solid). ^1^H NMR (400 MHz, Chloroform-*d*) δ 7.99 (s, 1H), 7.56 (s, 1H), 7.31 (s, 1H), 7.26 (s, 1H), 4.13 (t, *J* = 8.7 Hz, 2H), 3.29 (t, *J* = 8.7 Hz, 2H), 1.59 (s, 9H). ^13^C NMR (100 MHz, Chloroform-*d*) δ 152.16, 150.87, 149.43, 144.63, 131.22 (q, *J* = 31.1 Hz), 130.66, 124.58, 123.95 (q, *J* = 270.7 Hz), 115.78 (q, *J* = 3.3 Hz), 111.22 (q, *J* = 3.8 Hz), 81.55, 47.81, 28.34, 28.02. LCMS (ESI): calcd for C_17_H_18_F_3_N_2_O_3_^+^ (M + H^+^): 355.1, found 355.2.

#### Synthesis of (3-Fluoroazetidin-1-yl)(4-(oxazol-5-yl)-6-(trifluoromethyl)indolin-1-yl)methanone (**5**)

To a solution of 4-(oxazol-5-yl)-6-(trifluoromethyl)indoline-1-carboxylate **3** (90 mg, 0.25 mmol, 1 equiv) in dichloromethane (5 mL), trifluoroacetic acid (300 mg, 2.5 mmol, 10 equiv) was added dropwise and stirred at room temperature for 2 h. After that, the mixture was concentrated to give crude 5-(6-(trifluoromethyl)indolin-4-yl)oxazole **4** (65 mg, yellow solid).

To a solution of 5-(6-(trifluoromethyl) indolin-4-yl) oxazole **4** (65 mg, 0.25 mmol, 1.0 equiv) and 3-fluoroazetidine-1-carbonyl chloride (58 mg, 0.43 mmol, 1.7 equiv) in dichloromethane (5 mL) at room temperature was added triethylamine (76 mg, 0.75 mmol, 3 equiv), and the mixture stirred for 16 h. After that, the mixture was concentrated and purified by column chromatography to afford (3-fluoroazetidin-1-yl)(4-(oxazol-5-yl)-6-(trifluoromethyl)indolin-1-yl)methanone **5** (10 mg, 11% yield over two steps, white solid). ^1^H NMR (600 MHz, Chloroform-*d*) δ 8.01 (s, 2H), 7.58 (s, 1H), 7.33 (s, 1H), 5.37 (dm, *J* = 57.0 Hz, 1H), 4.41 (m, 2H), 4.26 (m, 2H), 4.08 (t, *J* = 8.5 Hz, 2H), 3.37 (t, *J* = 8.5 Hz, 2H). ^13^C NMR (150 MHz, Chloroform-*d*) δ 158.33 (d, *J* = 1.4 Hz), 150.79, 149.33, 145.33, 131.07 (q, *J* = 32.5 Hz), 130.18, 124.49, 124.25, 123.87 (q, *J* = 271.5 Hz), 116.10 (q, *J* = 4.0 Hz), 112.32 (q, *J* = 3.8 Hz), 82.10 (d, *J* = 204.0 Hz), 58.21 (d, *J* = 25.5 Hz), 48.39, 29.08. HRMS (APCI): exact mass calcd for C_16_H_14_F_4_N_3_O_2_^+^ (M + H^+^): 356.1017, found 356.1015.

#### Synthesis of 1-(4-(Oxazol-5-yl)-6-(trifluoromethyl)indoline-1-carbonyl)azetidin-3-yl 4-Nitrobenzenesulfonate (**6**)

To a solution of triphosgene (23.3 mg, 0.079 mmol, 0.4 equiv) in dichloromethane (3 mL) at 0 °C, 5-(6-(trifluoromethyl)indolin-4-yl)oxazole **4** (50 mg, 0.197 mmol, 1.0 equiv) in dichloromethane (5 mL) was added dropwise and stirred at room temperature for 2 h. Then, the mixture was added dropwise to a solution of Et_3_N (100 mg, 0.985 mmol, 5 equiv) and azetidin-3-ol hydrochloride (86 mg, 0.788 mmol, 4 equiv) in dichloromethane at 0 °C. The resulting mixture was stirred at room temperature for 1 h. Then, the solvent was removed, and the residue was dissolved in ethyl acetate, washed with water and brine, dried over Na_2_SO_4_, and concentrated to give crude (3-hydroxyazetidin-1-yl)(4-(oxazol-5-yl)-6-(trifluoromethyl)indolin-1-yl)methanone (60 mg, 86%).

To a solution of (3-hydroxyazetidin-1-yl)(4-(oxazol-5-yl)-6-(trifluoromethyl)indolin-1-yl)methanone (22 mg, 0.062 mmol, 1 equiv) in dichloromethane at room temperature were added Et_3_N (18.8 mg, 0.186 mmol, 3.0 equiv), DMAP (0.15 mg, cat. 2%), and NsCl (20.7 mg, 0.093 mmol, 1.5 equiv) and stirred for 12 h. Then, the mixture was washed with water and brine, dried over Na_2_SO_4_, concentrated, and purified by Prep-TLC (elution with CH_2_Cl_2_/EtOAc (v/v = 91/9)) to give 1-(4-(oxazol-5-yl)-6-(trifluoromethyl)indoline-1-carbonyl)azetidin-3-yl 4-nitrobenzenesulfonate **6** (12 mg, 36%, white solid). ^1^H NMR (600 MHz, DMSO-d6) δ 8.56 (s, 1H), 8.49 (d, *J* = 9.0 Hz, 2H), 8.26 (d, *J* = 9.0 Hz, 2H), 7.95 (d, *J* = 1.2 Hz, 1H), 7.71 (s, 1H), 7.58 (d, *J* = 0.7 Hz, 1H), 5.31 (m, 1H), 4.38 (ddd, *J* = 10.0, 6.6, 0.8 Hz, 2H), 4.10 (ddd, *J* = 10.3, 3.8, 0.8 Hz, 1H), 4.01 (t, *J* = 8.7 Hz, 2H), 3.36 (t, *J* = 8.7 Hz, 2H). ^13^C NMR (150 MHz, DMSO-d6) δ 157.79, 152.77, 151.43, 148.54, 146.36, 140.85, 132.04, 129.99, 129.24 (q, *J* = 31.5 Hz), 125.61, 125.49, 125.43 (q, *J* = 270.0 Hz), 124.95, 115.13 (q, *J* = 3.6 Hz), 110.99 (q, *J* = 3.1 Hz), 70.77, 57.43, 48.22, 29.11. HRMS (APCI): exact mass calcd for C_22_H_18_F_3_N_4_O_7_S^+^ (M + H^+^): 539.0843, found 539.0860.

### Radiochemistry

An aliquot of [^18^F]fluoride (around 30 mCi, 1110 MBq) was added to tetraethylammonium bicarbonate (1 mg/mL in MeOH) in a V-vial sealed and heated at 110 °C with nitrogen gas. Dry acetonitrile (1 mL) was added when no liquid was left. This step was repeated twice. Then, precursor **6** (1 mg) in DMF/*t*BuOH (100/300 μL) was added, and the mixture was heated at 140 °C for 10 min. After that, the mixture was diluted with HPLC eluting buffer (MeCN/H_2_O, v/v = 40/60, 2 mL) and purified with a Phenomenex Luna 5 μm C18(2) 100 Å Prep Column (10 × 250 mm) with MeCN/H_2_O (v/v = 40/60, containing 0.1% NEt_3_).

### Measurement of log*D*

The lipophilicity was measured according to a previous report.^21^ In brief, [^18^F]**5**, *n*-octanol (3 mL), and PBS (3 mL) were added in a centrifugal tube, followed by vortexing for 3 min and centrifugation (∼14,000 rpm) for 5 min. Then, PBS (around 500 μL) and *n*-octanol (around 50 μL) were weighed and measured by a gamma counter.

### Stability of [^18^F]5 in Serum

Mouse serum (Sigma-Aldrich, cat. M5905, 0.4 mL), rat serum (Abcam, cat. ab7488, 0.4 mL), NHP serum (Abcam, cat. ab155109, 0.4 mL), and human serum (Sigma-Aldrich, cat. H4522, 0.4 mL) were incubated at 37 °C for 5 min. Then, formulated [^18^F]**5** was added and incubated at 37 °C (Benfluorex was used in the positive control test). At 5, 30, and 60 min after incubation with [^18^F]**5**, 100 μL of the samples was drawn in centrifugal tubes and added to ice-cold MeCN (100 μL). The mixtures were centrifuged at 10,000*g* for 5 min, and the supernatants were analyzed by radio-HPLC.

### In Vitro Autoradiography

The autoradiography study was conducted as previously reported.^22,23^ In brief, sagittal rat brain sections (20 μm) were incubated with Tris–HCl buffer (50 mM) at room temperature for 20 min and then incubated with [^18^F]**5** (1 μCi/mL). In the blocking study, the brain sections were incubated with [^18^F]**5** in the presence of Soticlestat, CHL2205, or **5** (10 μM). Then, the brain sections were washed with ice-cold Tris–HCl buffer (50 mM) for 2 min and cold distilled water for 10 s. Subsequently, the brain sections were dried, positioned on an imaging screen, and scanned by an Amersham Typhoon analyzer system.

### PET Imaging

The dynamic PET imaging study was performed in SD rats as previously reported.^21^ A Genisys G8 PET scanner (Sofie Biosciences, USA) was used for dynamic PET imaging in rats with [^18^F]**5** (0.04 mCi) for 60 min. In the blocking study, rats were pretreated with Soticlestat (1 mg/kg, intravenous injection via the tail vein) 10 min before administration of [^18^F]**5**. For the chase study, the rats were treated with Soticlestat (1 mg/kg, i.v.) 20 min after administration of [^18^F]**5**.

### Whole-Body Biodistribution

The biodistribution study was conducted in CD-1 mice as previously reported.^24^ CD-1 mice were administrated with [^18^F]**5** (10 μCi) and divided into four groups and then euthanized at 5, 15, 30, and 60 min after administration of [^18^F]**5**. Major organs were collected, weighed, and measured by a gamma counter.

### Radiometabolite Analysis

Radiometabolite analysis of [^18^F]**5** was conducted as previously reported.^25^ SD rats were administrated with [^18^F]**5** and then euthanized at 20 and 60 min after administration of [^18^F]**5**. The brains were collected, homogenized, and centrifuged with cold acetonitrile and PBS buffer. Then, the supernatants were injected into a radio-HPLC with unlabeled **5** as an internal standard. Both [^18^F]**5** and its ^18^F-metabolites were collected by HPLC and then measured by a gamma counter. The same procedure was performed for the plasma.

## Supporting Information Available

Synthesis of precursor; stability tests; time-activity curves; whole-body biodistribution; HPLC radio-chromatograms; and ^1^H and ^13^C NMR spectra (PDF)

## Abbreviations

AD: Alzheimer’s disease
BBB: blood–brain barrier
CYP46A1: cholesterol 24-hydroxylase
CNS: central nervous system
PET: positron emission tomography
PD: Parkinson’s disease
RCY: radiochemical yield

## References

[ref1] Luo J.; Yang H.; Song B.-L. Mechanisms and regulation of cholesterol homeostasis. Nat. Rev. Mol. Cell Biol. 2020, 21 (4), 225–245. 10.1038/s41580-019-0190-7. 31848472

[ref2] Björkhem I.; Lütjohann D.; Breuer O.; Sakinis A.; Wennmalm Å. Importance of a Novel Oxidative Mechanism for Elimination of Brain Cholesterol: TURNOVER OF CHOLESTEROL AND 24(*S*)-HYDROXYCHOLESTEROL IN RAT BRAIN AS MEASURED WITH ^18^O_2_ TECHNIQUES IN VIVO AND IN VITRO*. J. Biol. Chem. 1997, 272 (48), 30178–30184. 10.1074/jbc.272.48.30178. 9374499

[ref3] Dietschy J. M.; Turley S. D. Thematic review series: Brain Lipids. Cholesterol metabolism in the central nervous system during early development and in the mature animal. J. Lipid Res. 2004, 45 (8), 1375–1397. 10.1194/jlr.R400004-JLR200. 15254070

[ref4] Russell D. W.; Halford R. W.; Ramirez D. M. O.; Shah R.; Kotti T. Cholesterol 24-Hydroxylase: An Enzyme of Cholesterol Turnover in the Brain. Annu. Rev. Biochem. 2009, 78, 1017–1040. 10.1146/annurev.biochem.78.072407.103859. 19489738 PMC2837268

[ref5] Zhang J.; Liu Q. Cholesterol metabolism and homeostasis in the brain. Protein & Cell 2015, 6 (4), 254–264. 10.1007/s13238-014-0131-3. 25682154 PMC4383754

[ref6] Moutinho M.; Nunes M. J.; Rodrigues E. Cholesterol 24-hydroxylase: Brain cholesterol metabolism and beyond. Biochim. Biophys. Acta, Mol. Cell Biol. Lipids 2016, 1861 (12, Part A), 1911–1920. 10.1016/j.bbalip.2016.09.011. 27663182

[ref7] Katsuno M.; Adachi H.; Sobue G. Getting a handle on Huntington’s disease: the case for cholesterol. Nature Medicine 2009, 15 (3), 253–254. 10.1038/nm0309-253. 19265827

[ref8] Karasinska J. M.; Hayden M. R. Cholesterol metabolism in Huntington disease. Nature Reviews Neurology 2011, 7 (10), 561–572. 10.1038/nrneurol.2011.132. 21894212

[ref9] Djelti F.; Braudeau J.; Hudry E.; Dhenain M.; Varin J.; Bièche I.; Marquer C.; Chali F.; Ayciriex S.; Auzeil N.; Alves S.; Langui D.; Potier M.-C.; Laprevote O.; Vidaud M.; Duyckaerts C.; Miles R.; Aubourg P.; Cartier N. CYP46A1 inhibition, brain cholesterol accumulation and neurodegeneration pave the way for Alzheimer’s disease. Brain 2015, 138 (8), 2383–2398. 10.1093/brain/awv166. 26141492

[ref10] Dai L.; Zou L.; Meng L.; Qiang G.; Yan M.; Zhang Z. Cholesterol Metabolism in Neurodegenerative Diseases: Molecular Mechanisms and Therapeutic Targets. Molecular Neurobiology 2021, 58 (5), 2183–2201. 10.1007/s12035-020-02232-6. 33411241

[ref11] Duan Y.; Gong K.; Xu S.; Zhang F.; Meng X.; Han J. Regulation of cholesterol homeostasis in health and diseases: from mechanisms to targeted therapeutics. Signal Transduction and Targeted Therapy 2022, 7 (1), 265 10.1038/s41392-022-01125-5. 35918332 PMC9344793

[ref12] Krishnan H. S.; Ma L.; Vasdev N.; Liang S. H. 18F-Labeling of Sensitive Biomolecules for Positron Emission Tomography. *Chemistry – A*. European Journal 2017, 23 (62), 15553–15577. 10.1002/chem.201701581. PMC5675832 28704575

[ref13] Deng X.; Rong J.; Wang L.; Vasdev N.; Zhang L.; Josephson L.; Liang S. H. Chemistry for Positron Emission Tomography: Recent Advances in ^11^C-, ^18^F-, ^13^N-, and ^15^O-Labeling Reactions. Angew. Chem., Int. Ed. 2019, 58 (9), 2580–2605. 10.1002/anie.201805501. PMC6405341 30054961

[ref14] Rong J.; Haider A.; Jeppesen T. E.; Josephson L.; Liang S. H. Radiochemistry for positron emission tomography. Nat. Commun. 2023, 14 (1), 3257 10.1038/s41467-023-36377-4. 37277339 PMC10241151

[ref15] Chen Z.; Chen J.; Mast N.; Rong J.; Deng X.; Shao T.; Fu H.; Yu Q.; Sun J.; Shao Y.; Josephson L.; Collier T. L.; Pikuleva I.; Liang S. H. Synthesis and pharmacokinetic study of a 11C-labeled cholesterol 24-hydroxylase inhibitor using ‘in-loop’ [11C]CO2 fixation method. Bioorg. Med. Chem. Lett. 2020, 30 (9), 127068 10.1016/j.bmcl.2020.127068. 32178974 PMC7196435

[ref16] Koike T.; Constantinescu C. C.; Ikeda S.; Nishi T.; Sunahara E.; Miyamoto M.; Cole P.; Barret O.; Alagille D.; Papin C.; Morley T.; Fowles K.; Seibyl J.; Tamagnan G.; Kuroita T. Preclinical characterization of [18F]T-008, a novel PET imaging radioligand for cholesterol 24-hydroxylase. European Journal of Nuclear Medicine and Molecular Imaging 2022, 49 (4), 1148–1156. 10.1007/s00259-021-05565-z. 34651220 PMC8921165

[ref17] Constantinescu C. C.; Brown T.; Wang S.; Yin W.; Barret O.; Jennings D.; Tauscher J. Clinical Characterization of [18F]T-008, a Cholesterol 24-Hydroxylase PET Ligand: Dosimetry, Kinetic Modeling, Variability, and Soticlestat Occupancy. J. Nucl. Med. 2023, 64 (12), 1972 10.2967/jnumed.123.265912. 37770111 PMC10690114

[ref18] Haider A.; Zhao C.; Wang L.; Xiao Z.; Rong J.; Xia X.; Chen Z.; Pfister S. K.; Mast N.; Yutuc E.; Chen J.; Li Y.; Shao T.; Warnock G. I.; Dawoud A.; Connors T. R.; Oakley D. H.; Wei H.; Wang J.; Zheng Z.; Xu H.; Davenport A. T.; Daunais J. B.; Van R. S.; Shao Y.; Wang Y.; Zhang M.-R.; Gebhard C.; Pikuleva I.; Levey A. I.; Griffiths W. J.; Liang S. H. Assessment of cholesterol homeostasis in the living human brain. Science Translational Medicine 2022, 14 (665), eadc9967 10.1126/scitranslmed.adc9967. 36197966 PMC9581941

[ref19] Koike T.; Yoshikawa M.; Nomura I.; Ito Y.; Kimura E.; Ando H.; Hasui T.; Nishi T. Heterocyclic compound, 2014.

[ref20] OECD Test No. 107: Partition Coefficient (n-octanol/water): Shake Flask Method, 1995.

[ref21] Rong J.; Mori W.; Xia X.; Schafroth M. A.; Zhao C.; Van R. S.; Yamasaki T.; Chen J.; Xiao Z.; Haider A.; Ogasawara D.; Hiraishi A.; Shao T.; Zhang Y.; Chen Z.; Pang F.; Hu K.; Xie L.; Fujinaga M.; Kumata K.; Gou Y.; Fang Y.; Gu S.; Wei H.; Bao L.; Xu H.; Collier T. L.; Shao Y.; Carson R. E.; Cravatt B. F.; Wang L.; Zhang M.-R.; Liang S. H. Novel Reversible-Binding PET Ligands for Imaging Monoacylglycerol Lipase Based on the Piperazinyl Azetidine Scaffold. J. Med. Chem. 2021, 64 (19), 14283–14298. 10.1021/acs.jmedchem.1c00747. 34569803 PMC9090218

[ref22] Shao T.; Chen Z.; Rong J.; Belov V.; Chen J.; Jeyarajan A.; Deng X.; Fu H.; Yu Q.; Rwema S. H.; Lin W.; Papisov M.; Josephson L.; Chung R. T.; Liang S. H. [18F]MAGL-4–11 positron emission tomography molecular imaging of monoacylglycerol lipase changes in preclinical liver fibrosis models. Acta Pharmaceutica Sinica B 2022, 12 (1), 308–315. 10.1016/j.apsb.2021.07.007. 35127387 PMC8799882

[ref23] Rong J.; Yamasaki T.; Li Y.; Kumata K.; Zhao C.; Haider A.; Chen J.; Xiao Z.; Fujinaga M.; Hu K.; Mori W.; Zhang Y.; Xie L.; Zhou X.; Collier T. L.; Zhang M.-R.; Liang S. Development of Novel ^11^C-Labeled Selective Orexin-2 Receptor Radioligands for Positron Emission Tomography Imaging. ACS Med. Chem. Lett. 2023, 14 (10), 1419–1426. 10.1021/acsmedchemlett.3c00320. 37849554 PMC10577698

[ref24] Rong J.; Zhao C.; Xia X.; Li G.; Haider A.; Wei H.; Chen J.; Xiao Z.; Li Y.; Zhou X.; Xu H.; Collier T. L.; Wang L.; Liang S. H. Evaluation of [18F]Favipiravir in Rodents and Nonhuman Primates (NHP) with Positron Emission Tomography. Pharmaceuticals 2023, 16 (4), 524 10.3390/ph16040524. 37111280 PMC10146102

[ref25] Rong J.; Yamasaki T.; Chen J.; Kumata K.; Zhao C.; Fujinaga M.; Hu K.; Mori W.; Zhang Y.; Xie L.; Chaudhary A. F.; Zhou X.; Zhang W.; Gao Y.; Zhang K.; Patel J. S.; Song Z.; Collier T. L.; Yuan H.; Ran C.; Haider A.; Li Y.; Zhang M.-R.; Liang S. Development of a Candidate 11C-Labeled Selective Phosphodiesterase 1 Radioligand for Positron Emission Tomography. ACS Omega 2024, 9 (44), 44154–44163. 10.1021/acsomega.4c03214. 39524622 PMC11541501

