# Supplementary material for: Development of a Novel 18F-Labeled Radioligand for Imaging Cholesterol 24-Hydroxylase with Positron Emission Tomography
Source: ACS Pharmacol Transl Sci. 2025 Feb 24;8(3):800–7. doi: 10.1021/acsptsci.4c00683 (PMC11915032; doi:10.1021/acsptsci.4c00683)

Supporting Information

Development of a novel ^{18}F-labeled radioligand for imaging cholesterol 24-hydroxylase with positron emission tomography

Jian Rong[a], Chunyu Zhao[a], Ahmad F. Chaudhary[a], Jiahui Chen[a], Xin Zhou[a], Kuo Zhang[a], Zhendong Song[a], Zhenkun Sun[b], Yabiao Gao[a], Zachary Zhang[a], Siyan Feng[a], Thomas Lee Collier[a], Hongjie Yuan[b], Jimmy S. Patel[a], Achi Haider[a], Yinlong Li[a], and Steven H. Liang[a,*]

[a] *Department of Radiology and Imaging Sciences, Emory University, Atlanta, Georgia 30322, United States*

[b] *Department of Pharmacology and Chemical Biology, Emory University School of Medicine, Atlanta, GA 30322, United States*

*Correspondence: steven.liang@emory.edu

Table of contents

Supporting Scheme, Figures, and Tables....3

Scheme S1 Synthesis of precursor **6**....3

Figure S1 Off-target pharmacological evaluation of compound **5**....3

Figure S2 Stability tests of [^{18}F]**5**....4

Figure S3 Time-activity-curves of [^{18}F]**5** in rat whole brain PET study under baseline and blocking conditions....4

Figure S4 The time-activity-curves of [^{18}F]**5** in chase study with Soticlestat in rat brain....5

Table S1 Whole-body biodistribution study of [^{18}F]**5** in CD-1 mice....5

Table S2 Optimization of ^{18}F-labeling conditions....6

HPLC radio-chromatograms....6

Radiometabolite analysis of [^{18}F]5 on rats....9

^{1}H NMR and ^{13}C NMR spectra....10

1 Supporting Scheme, Figures, and Tables

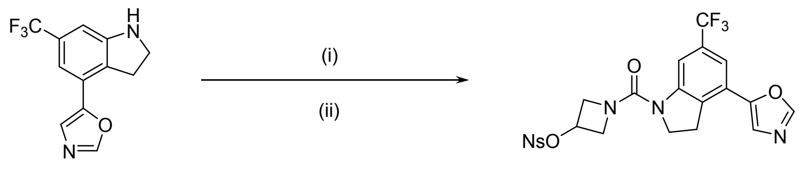

4 **6**

Scheme S1. Synthesis of precursor **6**. (i) azetidin-3-ol hydrochloride, triphosgene, NEt_3, dichloromethane, 0 °C to room temperature, 1 h; (ii) 4-nitrobenzenesulfonyl chloride, 4-(dimethylamino)pyridine, NEt_3, dichloromethane, room temperature, 12 h, 31% yield over two steps.

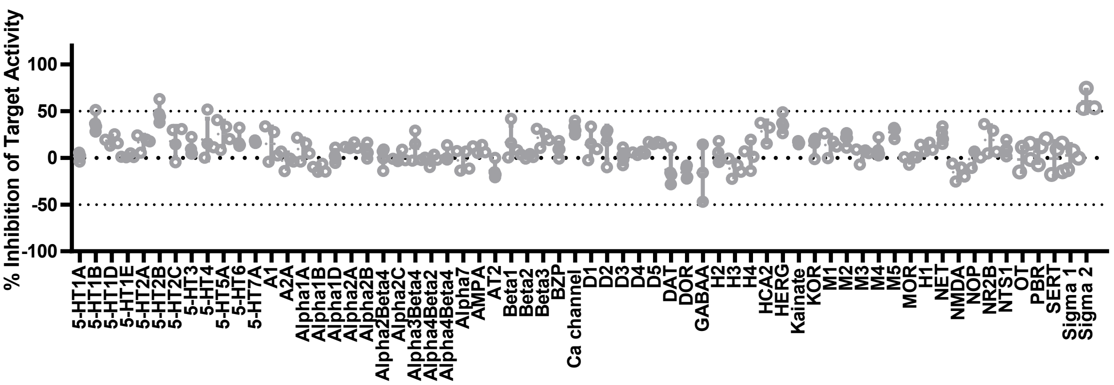

Figure S1. Off-target pharmacological evaluation of compound **5** against major CNS targets, including common GPCRs, enzymes, ion channels, and transporters: initial screening at a concentration of 10 µM (supported by the NIMH PDSP). All data are mean ± SD (n ≥ 3). No significant off-target binding (> 50%) was observed at 10 µM compound testing concentration, except sigma 2 receptor (Ki = 5421 nM).

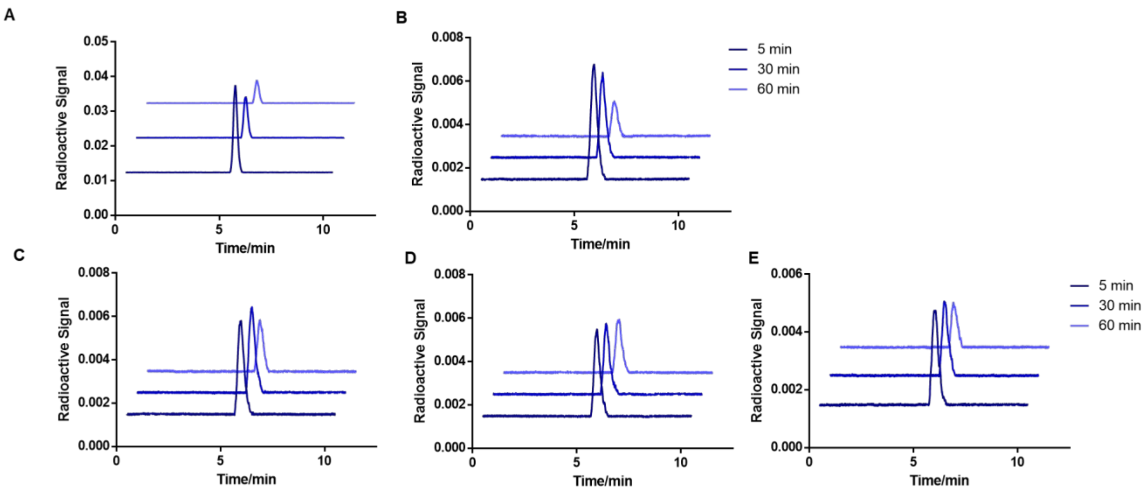

Figure S2. Stability tests of [^{18}F]**5**. (A) Stability of [^{18}F]**5** in saline; (B) Stability of [^{18}F]**5** in mouse serum; (C) Stability of [^{18}F]**5** in rat serum; (D) Stability of [^{18}F]**5** in NHP serum; (E) Stability of [^{18}F]**5** in human serum.

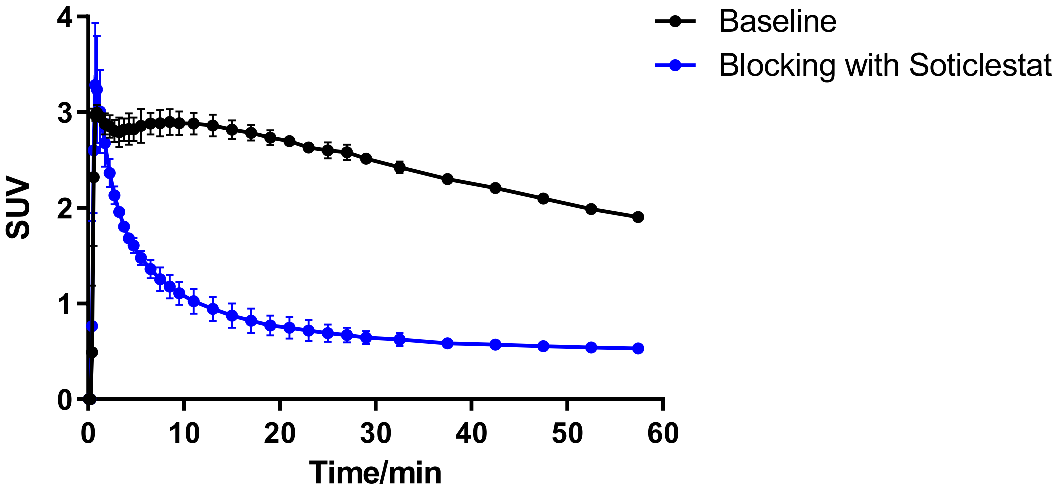

Figure S3. The time-activity-curves (0-60 min) of [^{18}F]**5** in rat whole brain PET study under baseline and blocking (Soticlestat, 1 mg/kg) conditions. All data were referred to as mean ± SD, n ≥ 3.

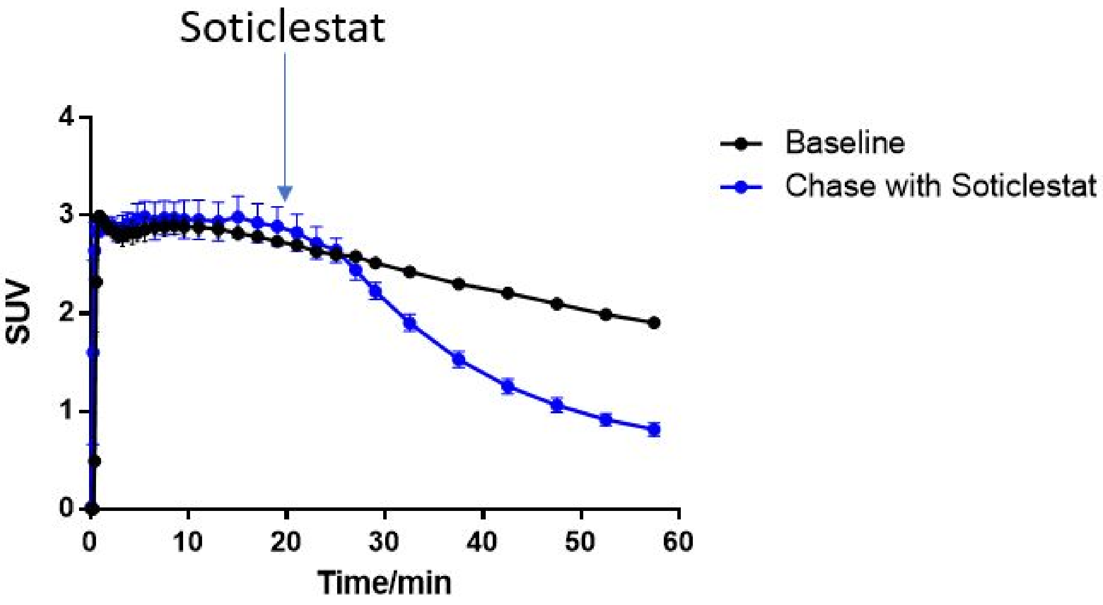

Figure S4. The time-activity-curves of [^{18}F]**5** in chase study with Soticlestat in rat brain. Soticlestat (1 mg/kg) was administrated at 20 min following tracer administration. All data are mean ± SEM (n ≥ 3).

Table S1. Whole-body *ex vivo* biodistribution study of [^{18}F]**5** in CD-1 mice.
Data are mean ± SD, n = 3

	5 min			15 min			30 min			60 min		
%ID/g	Mean	SD	N	Mean	SD	N	Mean	SD	N	Mean	SD	N
Brain	11.59	1.43	3	8.56	0.42	3	4.62	0.65	3	2.21	0.28	3
Blood	2.72	0.21	3	1.67	0.14	3	0.78	0.10	3	0.29	0.03	3
Muscle	1.81	0.27	3	0.86	0.14	3	0.47	0.09	3	0.22	0.06	3
Spleen	2.49	0.45	3	1.06	0.08	3	0.57	0.18	3	0.20	0.01	3
Heart	2.62	0.30	3	1.55	0.80	3	0.62	0.13	3	0.21	0.00	3
Lung	4.13	0.58	3	2.56	0.58	3	1.13	0.27	3	0.36	0.12	3
Pancreas	4.17	0.98	3	2.02	0.32	3	1.41	0.53	3	0.33	0.11	3
Stomach	1.10	0.28	3	0.77	0.06	3	0.60	0.31	3	0.53	0.29	3
Small Intestine	18.05	3.90	3	65.23	19.68	3	14.85	3.40	3	12.47	2.76	3
Kidney	9.29	1.51	3	6.88	1.93	3	2.21	0.61	3	0.80	0.17	3
Liver	30.73	4.46	3	19.53	1.67	3	10.01	1.44	3	6.50	0.68	3
Bone	2.63	0.96	3	2.23	0.60	3	1.75	0.32	3	2.44	0.57	3

Table S2. Optimization of ^{18}F-labeling conditions.

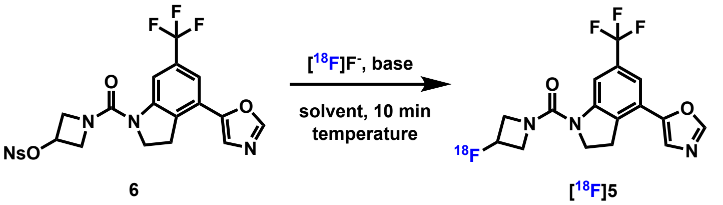

Entry	Base	Solvent	Temperature	RCC
1	K_2CO_3/K222	MeCN/*t*BuOH (3:1)	120 °C	2%
2	TEAB	MeCN/*t*BuOH (3:1)	120 °C	5%
3	TEAB	MeCN/*t*BuOH (1:3)	120 °C	2%
4	TEAB	*t*BuOH	120 °C	5%
5	TEAB	DMF/*t*BuOH (3:1)	120 °C	7%
6	TEAB	DMF/*t*BuOH (3:1)	140 °C	13%

Typical reaction conditions: **6** (1 mg), [^{18}F]F$^-$ (0.5-1 mCi), base, solvent, 10 min. RCC was determined by radio-TLC. TEAB = tetraethylammonium bicarbonate.

HPLC radio-chromatograms of [^{18}F]5

Semi-prep radio-HPLC chromatogram of [^{18}F]5

Column: Phenomenex Luna® 5 µm C18(2) 100 Å Prep Column (10 × 250 mm)

Mobile phase: CH_3CN-H_2O=40%-60% (containing 0.1% NEt_3)

flow rate: 5.0 mL/min

UV: 254 nm

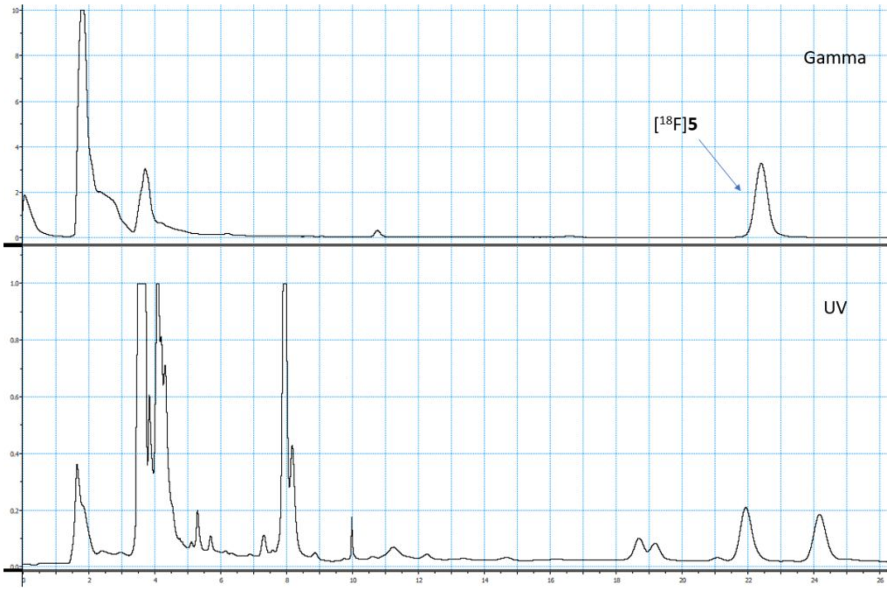

Analytical radio-HPLC chromatogram of [^{18}F]5

Column: XBridge C18 3.5 μm column (4.6 × 100 mm)

Mobile phase: CH_3CN-H_2O = 40%-60% (containing 0.1% NEt_3)

flow rate: 1.0 mL/min

UV: 254 nm

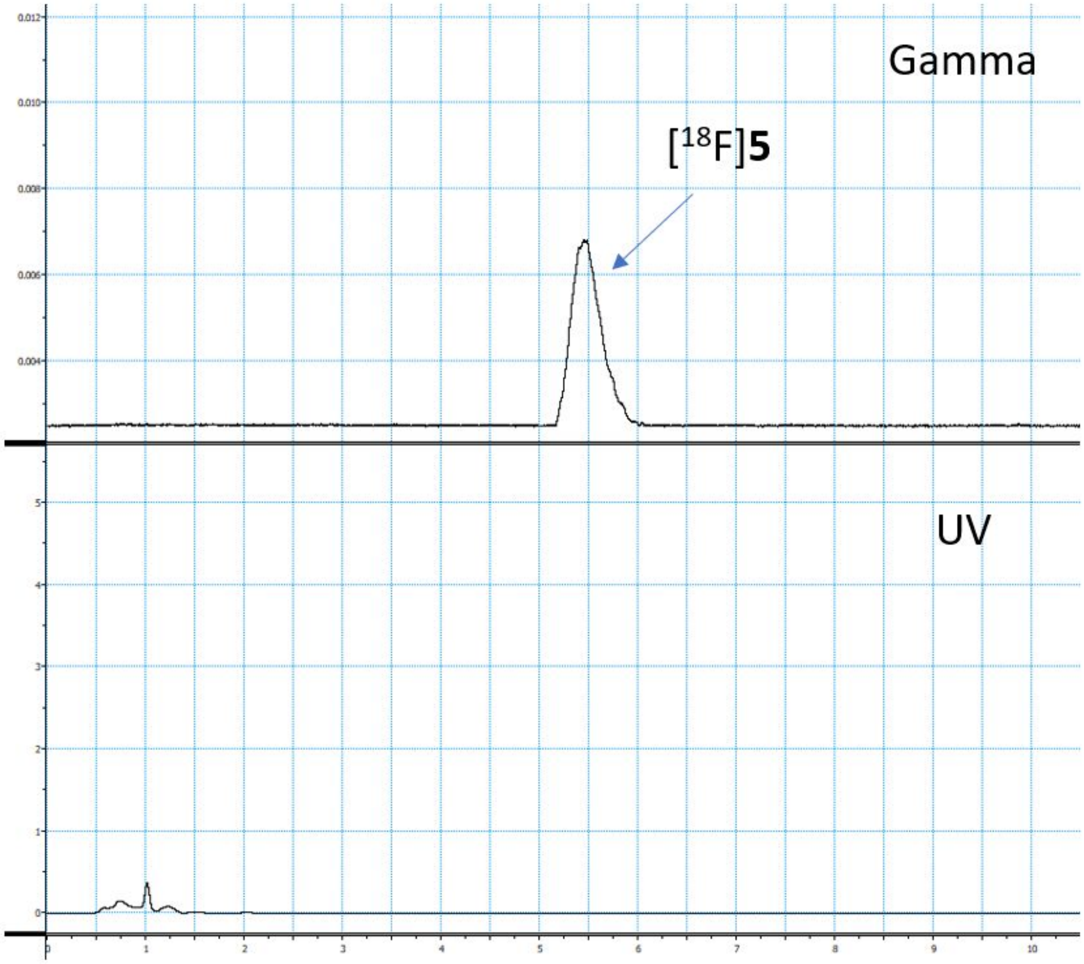

Co-injection of [^{18}F]5 with unlabeled 5

Column: XBridge C18 3.5 μm column (4.6 × 100 mm)

Mobile phase: CH_3CN-H_2O = 45%-55% (containing 0.1% NEt_3)

flow rate: 1.0 mL/min

UV: 254 nm

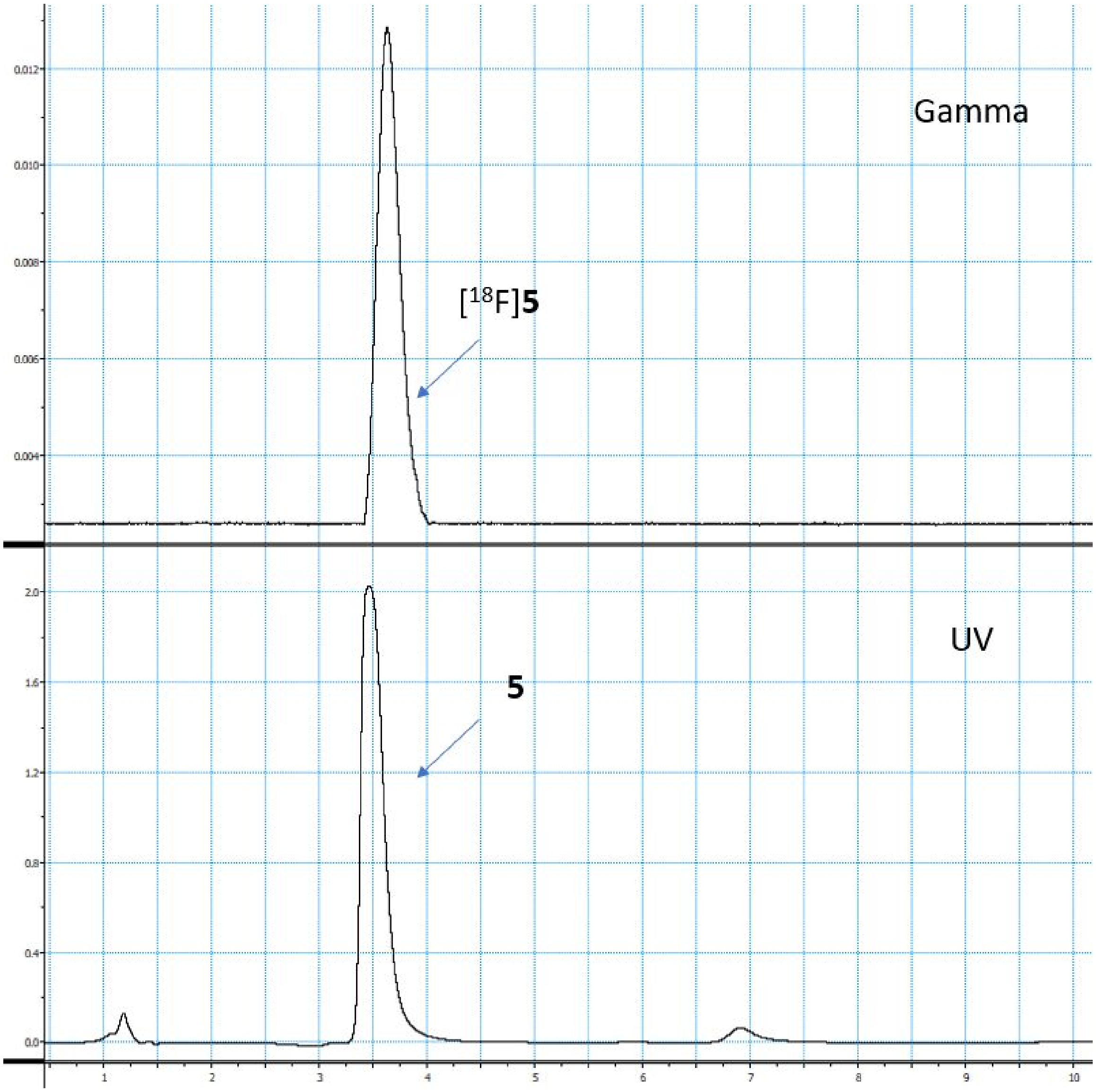

Radiometabolite analysis on rats: The supernatant samples for radiometabolite analysis were injected into HPLC and the elutes were collected and measured by a gamma counter. Radiometabolite analysis in rat brain and plasma at 20 min:

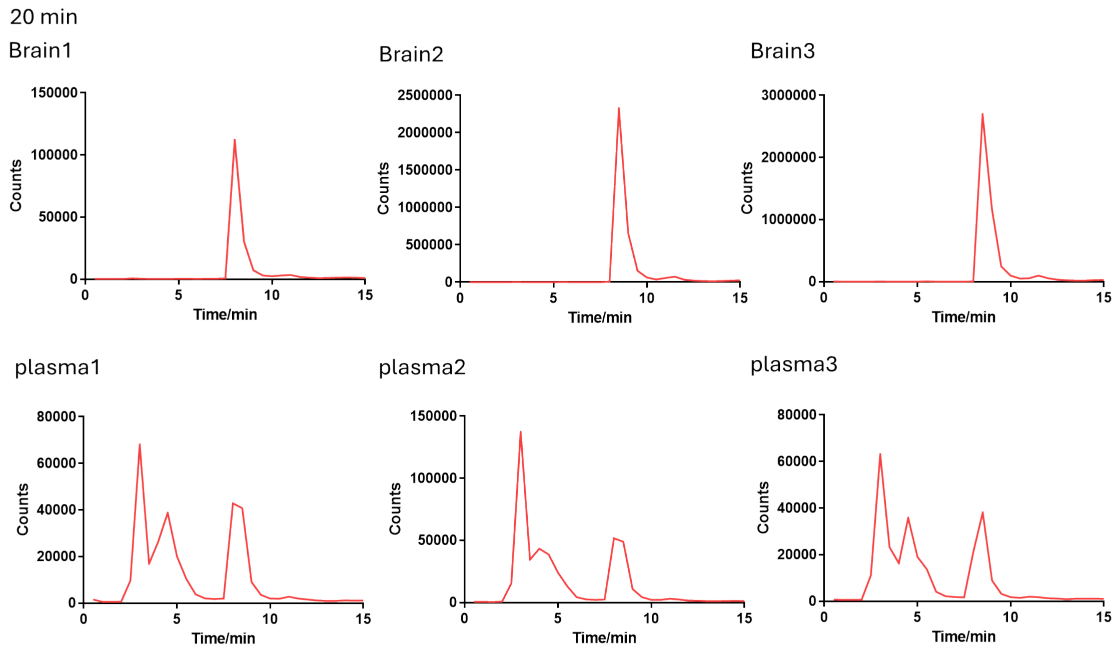

Radiometabolite analysis in rat brain and plasma at 60 min:

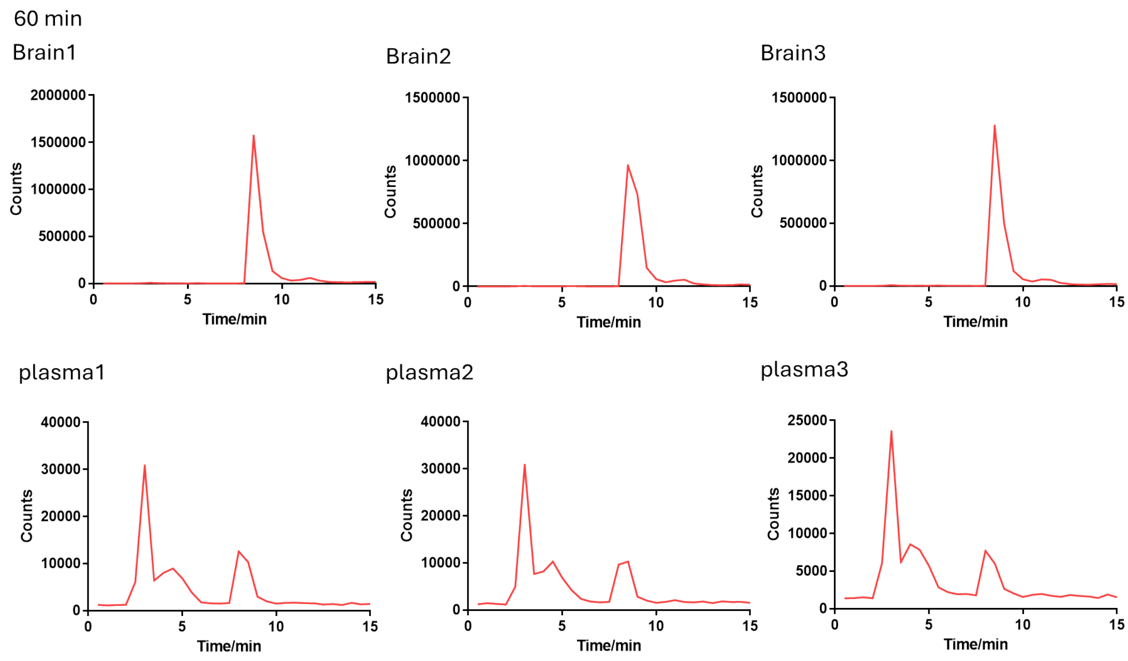

NMR spectra of isolated compounds

^{1}H NMR and ^{13}C NMR spectra of compound **2** ($CDCl_3$, 400 MHz)

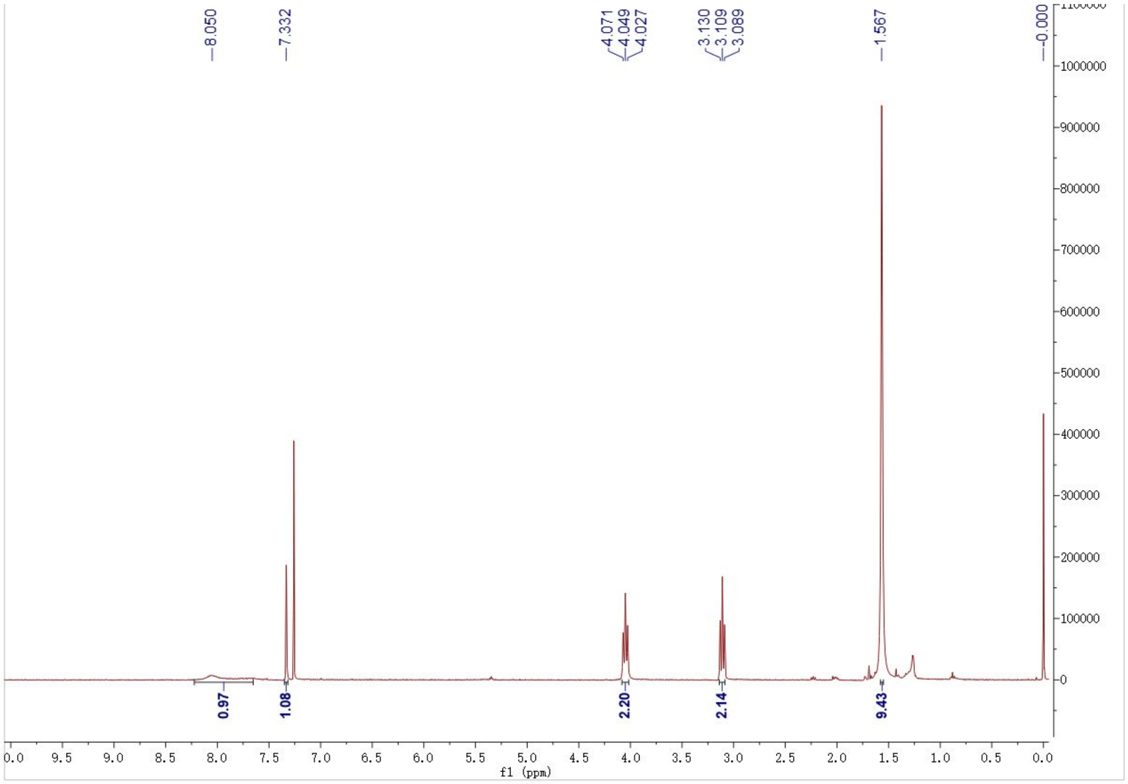

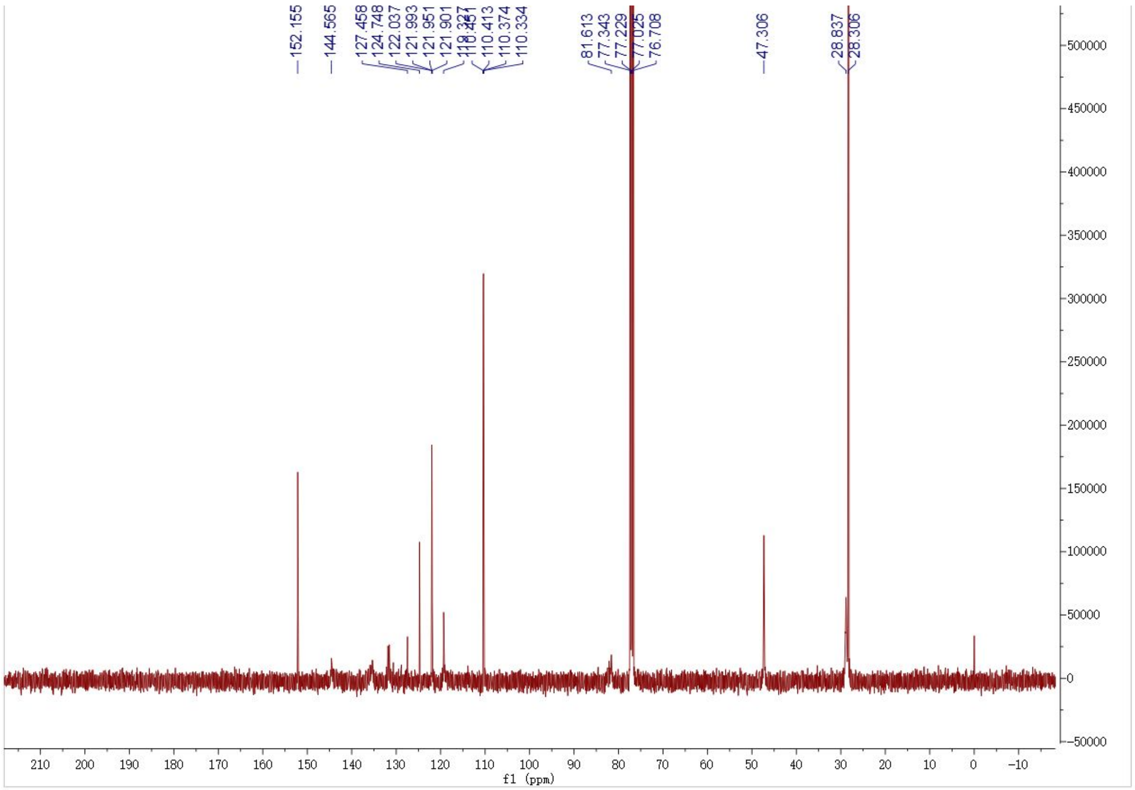

^{1}H NMR and ^{13}C NMR spectra of compound **3** ($CDCl_3$, 400 MHz)

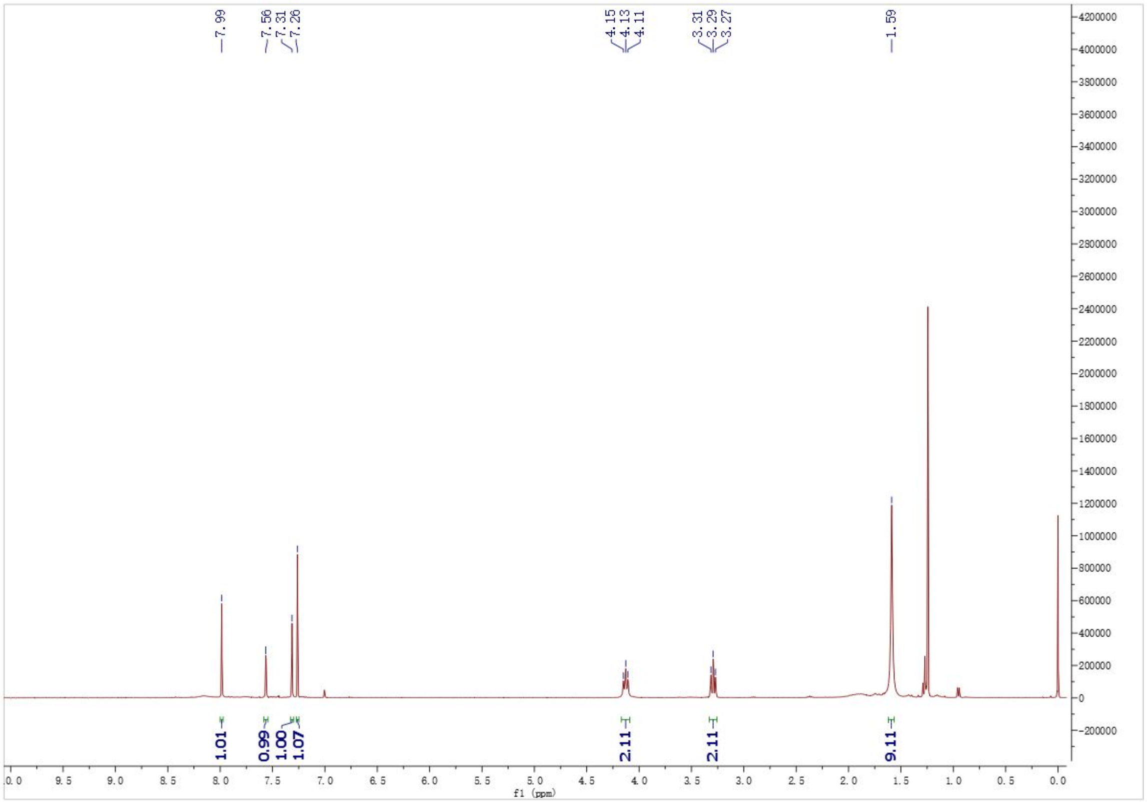

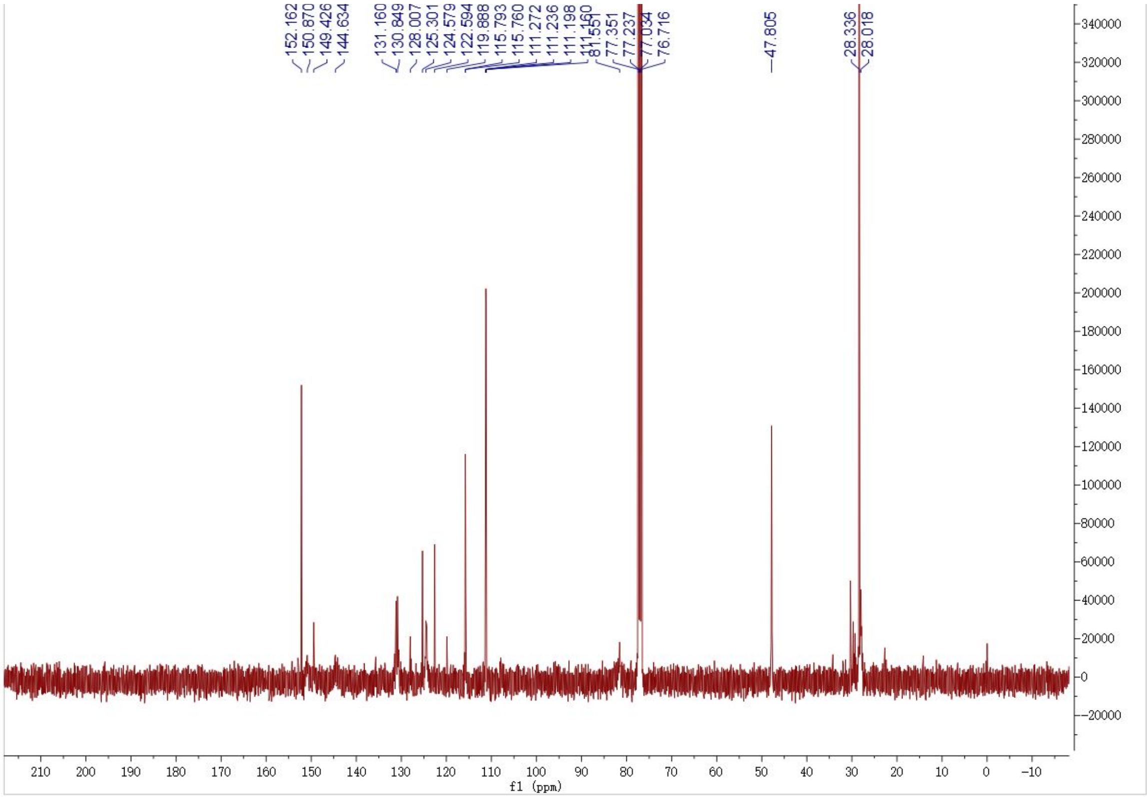

^1H NMR and ^{13}C NMR spectra of compound **5** ($CDCl_3$, 600 MHz)

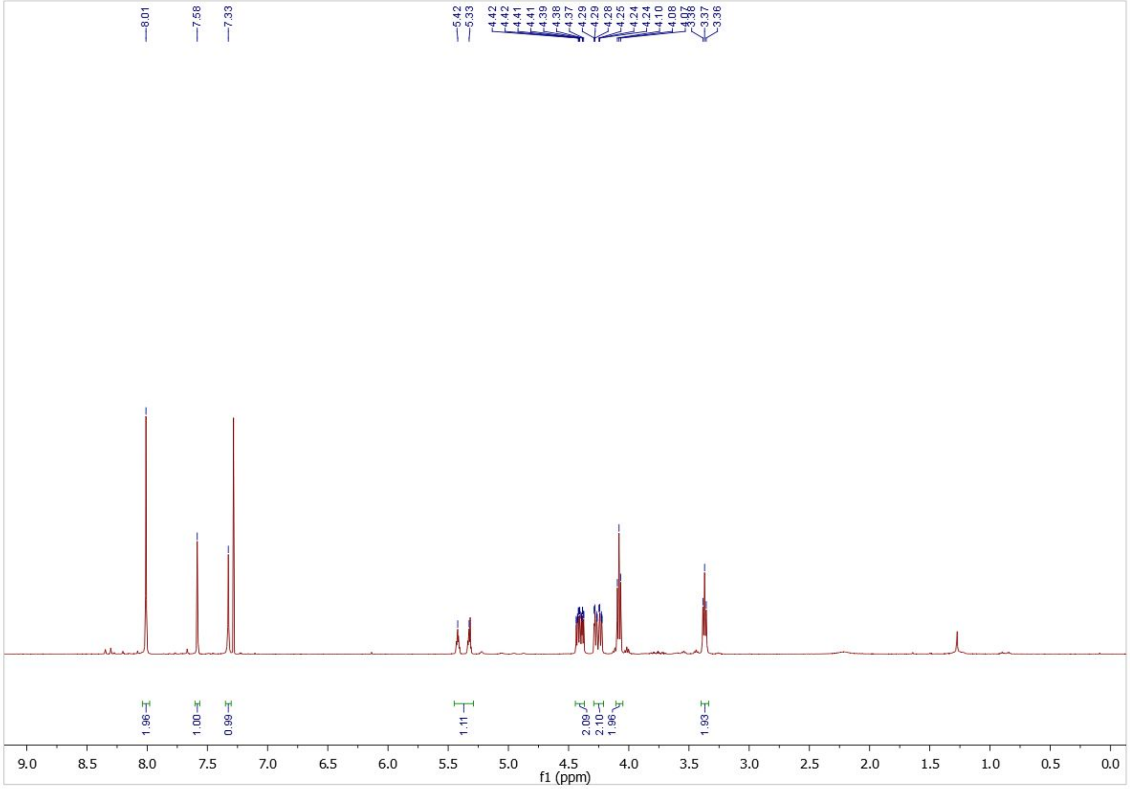

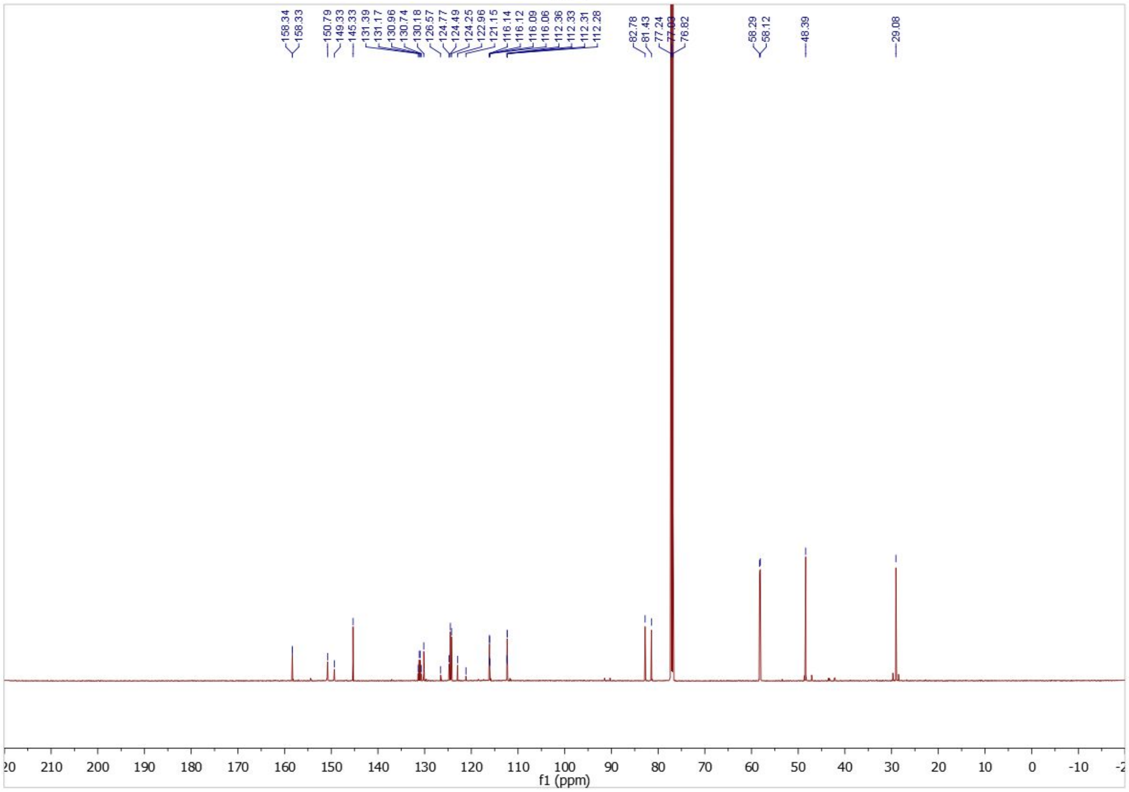

^{1}H NMR and ^{13}C NMR spectra of compound **6** (DMSO-d6, 600 MHz)

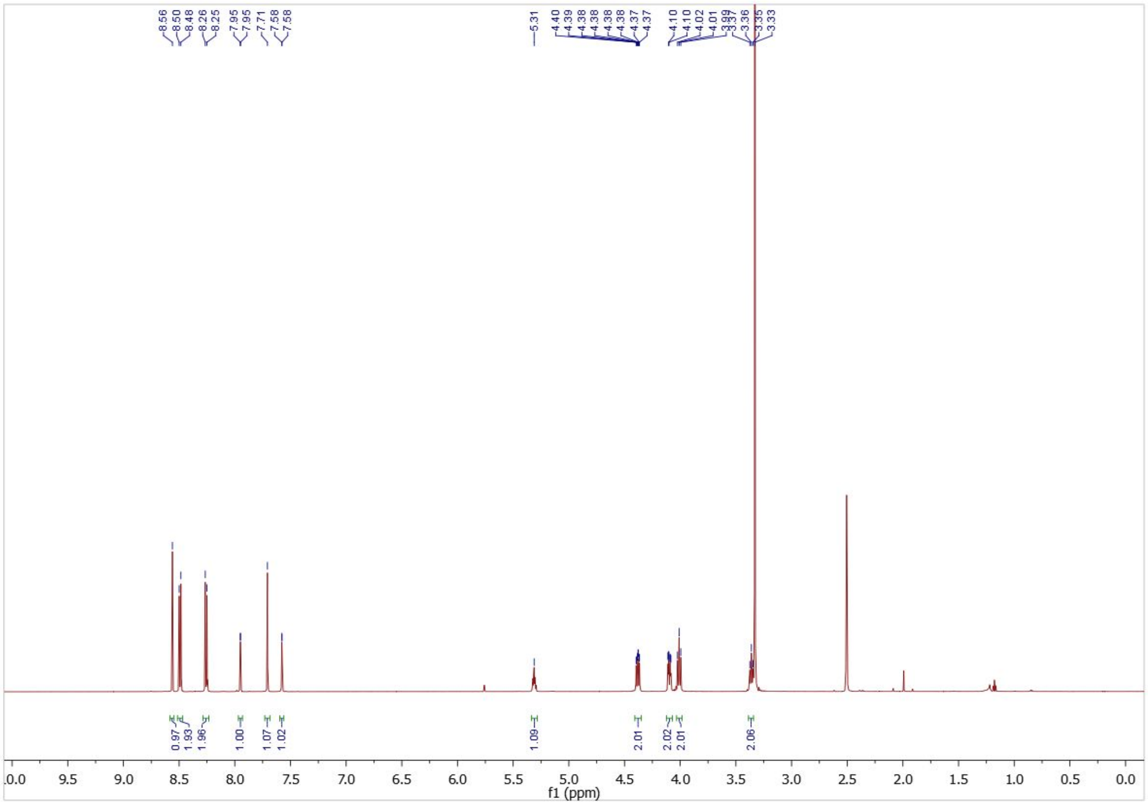

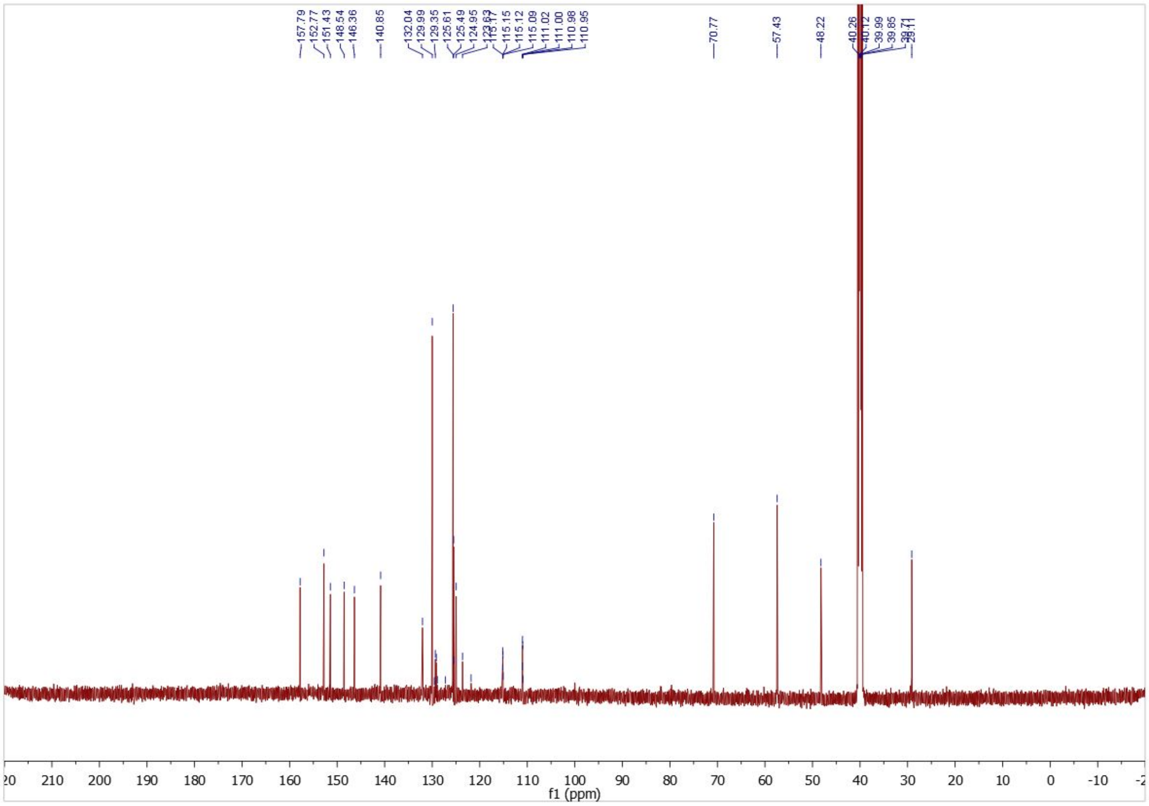

Supplement: Supplementary file 1 — pt4c00683_si_001.pdf [file pt4c00683_si_001.pdf]
